# Characterizing the Chemistry of One-Part Green Geopolymer Foams: The Role of Silica Fume and Fiber Hybridization

**DOI:** 10.1021/acsomega.4c10738

**Published:** 2025-05-14

**Authors:** Adem Ahıskalı, Barış Bayrak, Kenan Toklu, Oğuzhan Yavuz Bayraktar, Gökhan Kaplan, Abdulkadir Cüneyt Aydın

**Affiliations:** † Department of Civil Engineering, 187466 Kastamonu University, 37150 Kastamonu, Turkey; ‡ Department of Civil Engineering, 52975 Kafkas University, 36100 Kars, Turkey; § Department of Civil Engineering, Namık Kemal University, 59860 Tekirdağ/Çorlu, Turkey; ∥ Department of Civil Engineering, 37503 Atatürk University, 25030 Erzurum, Turkey

## Abstract

This study aims to investigate the mechanical, physical, durability and microstructure of slag-based and environmentally friendly foam geopolymer concrete with glass and polypropylene fiber hybridization using sustainable materials such as waste marble dust, silica fume (SF) and sodium metasilicate. One of the most critical advantages of geopolymer concrete compared to traditional Portland cement is its low carbon emissions. In the study, foam geopolymer concrete was produced using glass fiber (GF) and polypropylene fiber (PPF) additives and three different silica fume ratios (0%, 7.5%, 15%) and the effects of these additives on the workability (flow diameter), transfer performance (sorptivity), compressive strength, flexural strength, thermal conductivity, high-temperature performance and freeze–thaw resistance were investigated. The experimental results showed that the hybridization of GF and PPF significantly increased the compressive strength and flexural strength of samples without silica fume. However, high silica fume content negatively affected the mechanical properties by creating voids in the matrix. The flexural performance of geopolymer foams was significantly improved by fiber reinforcement. Mainly, improvements were observed in both peak load and displacement values of hybrid fiber geopolymer foams. In addition, GF and PPF admixtures increased the strength of concrete, especially after high temperatures of 200 and 400 °C. However, the compressive strength of samples exposed to high temperatures of 600 °C decreased. It was determined that the hybridization of GF and PPF increased the thermal insulation performance of foam geopolymer concrete. SEM analyses revealed that silica fume and fiber additives significantly affected the microstructure and mechanical strength of geopolymer foams. This study highlights that waste marble powder has the potential to be utilized in environmentally friendly geopolymer concrete and that the use of SF, GF and PPF additives at optimum rates is essential for the performance of foam geopolymer concrete.

## Graphical abstract

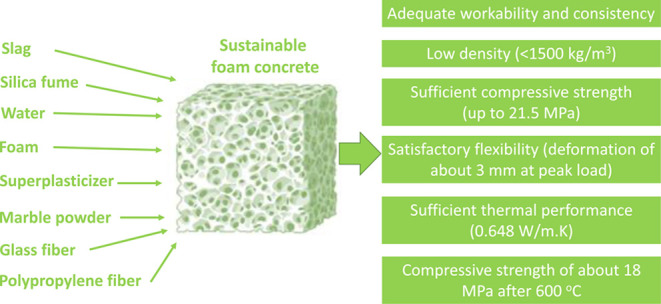

## 1 Introduction

Today, many scientific studies^1–5^ have tried to produce more sustainable concrete with reduced cement content to improve the strength of all kinds of concrete.^6^ It can be said that the most significant harm caused by cement production to the environment is the emission of harmful gases (especially greenhouse gases) into the atmosphere.^7^ Carbon dioxide produced due to the calcination of limestone clinkers used in cement production causes environmental pollution.^8–10^ Although it is stated in the literature that approximately 4100 million tons of cement are produced annually worldwide,^10,11^ considering that approximately 800 kg of CO_2_ is released into the atmosphere for the production of one ton of cement, the magnitude of the damage to the environment becomes apparent.^10,12,13^ To minimize these adverse effects, concrete is produced using a new binder material.^14^ The most important one is the geopolymer concrete invented by the French scientist Davidovits in 1970.^15–17^

Davidovits^16^ explained that geosynthesis is the science of producing artificial rock at temperatures below 100 °C to obtain the natural characteristics of the rock, such as longevity, heat stability and hardness.^18^ Although geopolymers can be considered mineral polymers obtained due to geosynthesis, they can be described as aluminosilicate-based binders.^18^ Nowadays, geopolymer (GC), a type of inorganic polymer group, is a binder that can potentially be a sustainable alternative to Portland cement and is becoming an increasingly popular research topic.^19–25^ GC is a type of concrete that is formed by activating aluminosilicate-based materials with various alkali activators, such as sodium hydroxide (NaOH) and sodium silicate (Na_2_SiO_3_).^26–29^ Alkaline and alkaline earth hydroxides, silicates, carbonates and sulfates are primarily used activators in the geopolymerization process and are often created with reagents provided by the market.^30–34^ Na_2_SiO_3_ and NaOH are alkaline activator solutions, the most critical components of the geopolymerization process, and are the most commonly used.^35^ For example, sodium silicate solution is obtained from the reaction of silica and sodium carbonate, prepared in specific proportions and calcined at high temperatures (1300–1400 °C) with water.^34,36^

Pozzolanic materials with high silicate and alumina values, which can react with alkaline solutions to produce aluminosilicate gel to bind aggregates in geopolymer concrete production, are widely used.^37,38^ Silica fume, a byproduct of the industry, is obtained by trapping the very fine particles in the gases coming out of the furnace while producing silicon metal or ferrosilicon alloys with the help of filters.^39^ The main reason why silica fume is widely used in the construction industry is its use as a filling material due to its very fine particles and its pozzolanic properties.^40^ Additionally, it is stated in the literature^41^ that silica fume, which has extraordinary physical and chemical properties, is more reactive than fly ash.^42^ In many scientific studies,^43,44^ it has been stated that using silica fume to produce geopolymer concrete improves the engineering properties of the concrete produced.^45^ Thokchom et al.^46^ indicated in their study that there were improvements in the porosity, compressive strength and sulfate resistance properties of the silica fume-substituted geopolymer mortars they produced. Another pozzolana commonly used in the production of geopolymer concrete is blast furnace slag. Blast furnace slag, whose chemical composition consists mainly of calcium, silica and alumina oxides, is a byproduct of the steel industry.^47,48^ There are many studies in the literature about slag-based geopolymer concrete.^49^ The silica and alumina contained in the slag, which is rich in calcium, dissolve and play an essential role in reducing the strength and curing temperature of the matrix of geopolymer concrete.^50–52^ Kumar et al.^53^ stated in their study that there were improvements in the setting times and compressive strength of the geopolymer concrete they produced by using fly ash and slag as alkaline activators. In addition, by using slag in the production of fly ash-based geopolymer concrete, the negative factors that occur during geopolymeration and affect the engineering properties of concrete can be reduced.^54^

To meet the increasing needs in the construction industry, studies are being carried out to make the products stronger and more durable in concrete technology.^55,56^ For this purpose, different types of fibers are used in concrete to improve its mechanical properties. While some studies on strengthening geopolymer concrete with poly­(vinyl alcohol), basalt and polyethylene fibers emphasize that the fibers form sufficient bonds with the concrete, causing limited cracks, it has been stated that in some concrete mixtures, the bonds are very weak and cause large cracks.^57–60^ For these reasons, many scientists are working on strengthening geopolymer concrete with glass fiber.^60–66^ Glass fiber, one of these fiber types, is a material with high strength and durability.^67^ Since glass fiber has high strength, especially in tensile strength, the concrete is resistant to fracture and deformation, so it is preferred in critical tensile-strength structures.^67^

Wastes that harm the environment have triggered scientists to do more work on recycling waste. Marble dust, one of the industrial wastes that produces mud and dust due to mining processing stages, negatively affects the environment and especially ecological life.^68^ Marble dust, an industrial byproduct produced in approximately 200 t worldwide, is formed from cutting marble and appears in different sizes and the form of mud.^69,70^ One ton of marble slurry is produced from 1 ton of marble used in the marble processing process, and approximately 45% of the marble sludge consists of water.^71^ It has been stated in the literature that successful results have been obtained in large-scale tests conducted for marble-based geopolymer concretes and that there are also increases in compressive strength when used as fine aggregate in concrete.^72,73^ Lim et al.^74^ used waste marble powder as aggregate in fly ash-based geopolymer concrete in their study and stated that the engineering properties of concrete increased by adding up to 50% marble powder aggregate to the mixture.^75^

This study produced geopolymer concrete containing silica fume, slag, and waste marble dust. Its engineering properties were examined to make more environmentally friendly concrete. This study aims to experimentally investigate the effects of silica fume ratio, glass and polypropylene fiber additives on mechanical, physical and durability parameters of slag-based foam geopolymer concrete consisting of waste marble dust and sodium metasilicate. The effects of hybridizing glass and polypropylene fibers with three different silica fume ratios (0%, 7.5% and 15%) on foam geopolymer concrete’s mechanical, physical and durability properties were investigated. Compressive strength and flexural strength were applied for the mechanical properties of geopolymer foam concrete, apparent porosity, water absorption and oven-dry density were used for physical properties, flow diameter for fresh properties, sorptivity for transport properties and thermal conductivity coefficient tests were applied for thermal performance. In addition, freeze–thaw and high-temperature tests were used to evaluate the durability parameters of foam geopolymer concrete. Compressive strength and mass loss results were assessed after high temperatures of 200 C, 400 C and 600 C. Moreover, SEM analysis results were examined to evaluate the microstructure.

## 2 Materials and Method

### 2.1 Raw Materials

In the production of geopolymer foams, ground blast furnace slag and silica fume were used as the binder phase. The chemistry of GBFS and SF is given in Table 1. The specific gravity of GBFS is 2.87, and the specific gravity of SF is 2.23. The grain size distribution of GBFS and SF was measured by the Malvern PANalytical MASTERSIZER 3000 device and is presented in Figure 1a. XRD patterns of GBFS and SF are presented in Figure 1b.

**Table 1:** GBFS and SF Chemistry (% by Weight)

material	CaO	SiO_2_	Al_2_O_3_	Fe_2_O_3_	MgO	Na_2_O	K_2_O	SO_3_	LOI
GBFS	37.9	31.2	14.1	1.4	11.4	0.6	0.9	1.2	0.9
SF	0.7	94.5	0.4	0.3	0.2	0.2	0.5	0.2	2.9
WMP	57.1	0.1	0.1	0.1	1.5	0.4	0.2	0.1	40.2

**Figure 1:**
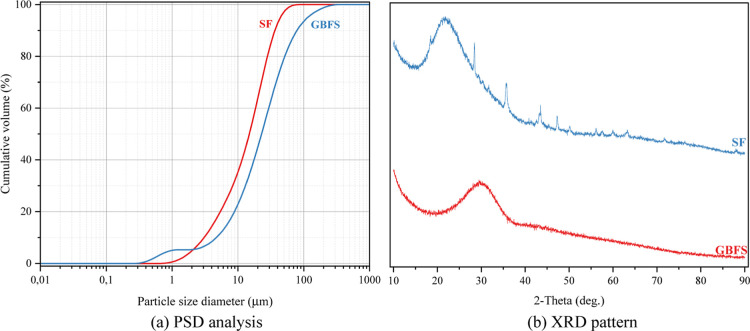
Physics and mineralogy of GBFS and SF.

Figure 1a shows that the grain size of SF is very fine. This results in SF having a high specific surface area and, therefore, high reactivity. The fine grains enable SF to create a filling effect in the matrix and improve the mechanical and durability properties through pozzolanic reactions. This feature may further increase SF’s water requirement and impair the geopolymer foam mixture’s workability. GBFS has a greater grain size range than SF. This distribution means GBFS can used as supplemental physical material because of its larger particles.

Due to its calcium content, GBFS generally contributes to forming C–S–H (calcium-silicate-hydrate) gels during alkaline activation. However, the wide distribution of grain size may pose difficulties in achieving a homogeneous reactivity. When used together, the fine structure of SF and the coarser grain structure of GBFS optimize different aspects of the matrix. The SF contributes microfilling and high reactivity on one side, while GBFS enhances the strength and durability of the matrix. SF is expected to help fill the voids, and at the same time, GBFS possesses a coarse particle structure.

In producing geopolymer foams, marble powder (WMP), another waste material, was used as an aggregate. The specific gravity of the marble powder is 2.67. Figure 2a shows the particle size distribution, and Figure 2b shows the XRD pattern.

**Figure 2:**
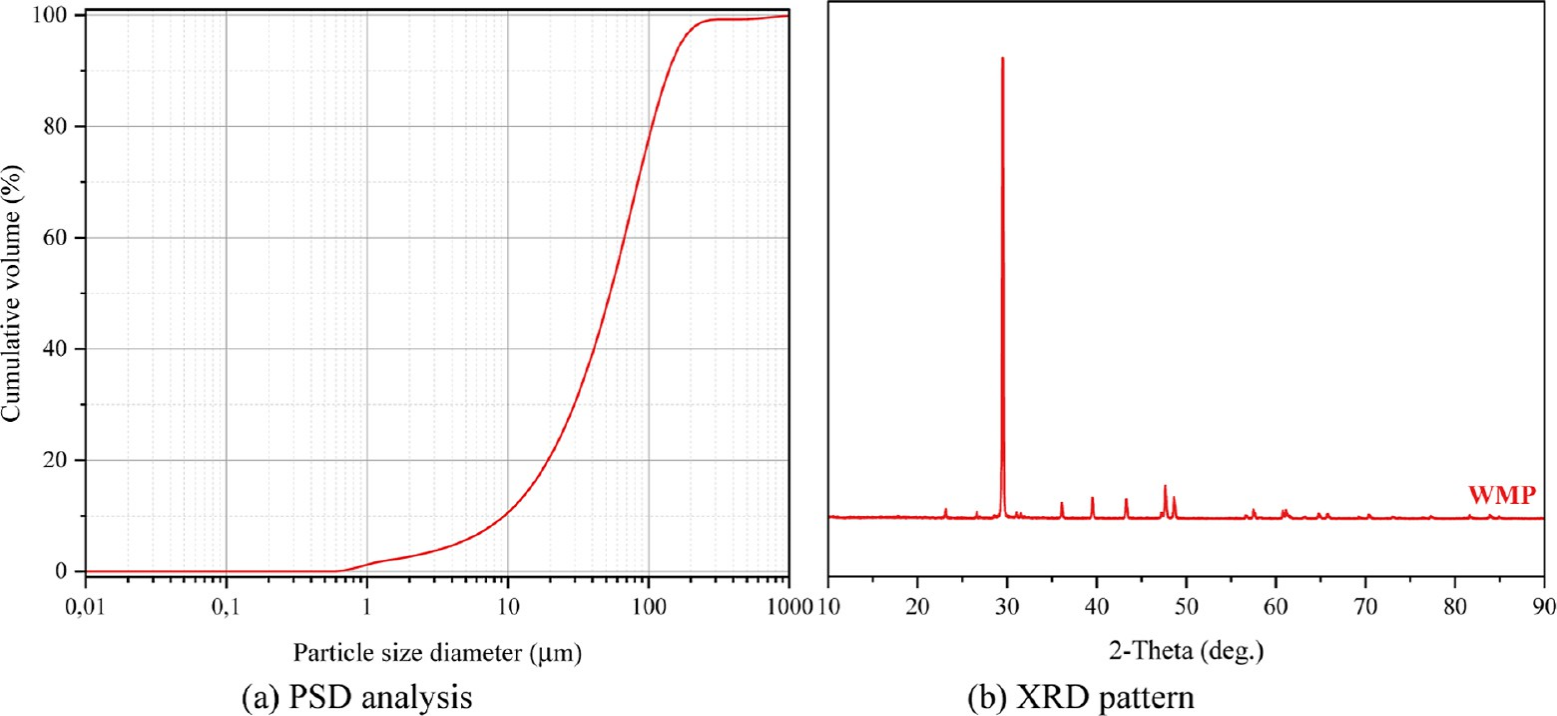
Physics and mineralogy of WMP.

The PSD of WMP shows a fine and relatively uniform distribution (Figure 2a). This indicates that the filling effect of the material can be high and effectively reduce voids in the matrix. The uniform distribution of grain sizes of WMP is essential for improving the mechanical strength and making the microstructure denser. Moreover, this property can improve the reaction efficiency, especially in materials used with alkaline activation (by nucleation effect). It was determined by PSD analysis that the marble powder grains were smaller than 750 μm. The peaks seen in the XRD pattern indicate that the mineralogical composition of WMP is dominated by the calcite (CaCO_3_) phase (Figure 2b). Calcite contains calcium carbonate as the main component of marble powder. The presence of other minerals such as low-intensity peaks, dolomite (CaMg­(CO_3_)_2_) or quartz (SiO_2_) was identified in the pattern. Such phases may affect the engineering properties of WMP. The sharp and clear XRD peaks show that WMP has a mostly crystalline structure. This indicates that the material has low reactivity and stable chemical behavior.

Sodium metasilicate (Na_2_SiO_3_) was used in powder form to activate GBFS and SF. These were used as binders to produce geopolymer foams. The specific gravity of sodium metasilicate is 2.61, and its molecular weight is 122.06 g/mol. Foam was added to the mix to reduce the unit weight and improve the thermal performance of the geopolymer samples. An organic resin-based foaming agent with air-entraining properties was used for this purpose. The foaming agent is liquid. However, its specific gravity is 1.12. Approximately 50 g of foaming agent was added to 1 L of water to obtain foam. A high-speed mixer (approximately 3300 rpm) was used to mix the foaming agent and water. A polycarboxylic ether-based water-reducing additive was added to the mixture to improve the workability of geopolymer foams. The specific gravity of this additive is 1.07 ± 2. The solid content is 37%. Glass (GF) and polypropylene fibers (PPF) were added to the mix to improve the geopolymer foams’ relatively low flexural strength and reduce the risk of shrinkage. These fibers are added to the mixture in mono or binary (hybrid) form. Table 2 shows the technical specifications of GF and PPF. These specifications given in Table 2 were obtained from the supplier companies.

**Table 2:** Technical Specifications of GF and PPF

properties	PPF	GF
diameter (mm)	12	12
length (μm)	18	18.5
Young modulus (GPa)	7	72
elongation (%)	25	4.6
tensile strength (MPa)	400	3450
specific gravity	0.91	2.56
melting point (°C)	165	840

### 2.2 Mixing Ratios and Preparation of Geopolymer Foams

The dosage of GBFS, the main binder in the production of geopolymer foams, is 700 kg/m^3^. SF replaced GBFS in 7.5% and 15% of cases. Sodium metasilicate (SM) was added to the mixture at 15 wt % of the binder phase consisting of GBFS and SF. In addition, a water-reducing additive (WRA/Based on polycarboxylic ether) of 4% of the weight of GBFS and SF was added to the mixture to ensure adequate workability. To reduce the unit weight of geopolymer foams, 15 kg/m^3^ of foam was added to the mixture. In this way, the unit weight of the mixtures will be reduced, and thus, the dead load in the structures will be reduced. GF and PPF were used in geopolymer foams at 2% by volume. In addition, these fibers were also used as hybrids at a rate of 2%. Mixing ratios and material quantities of geopolymer foams are given in Table 3.

**Table 3:** Mixing Ratios and Material Quantities (kg/m^3^) of Geopolymer Foams

mix IDs	GF (%)	PPF (%)	SF (%)	GBFS	SF	water	foam	WRA	WMP	SM	GF	PPF
0% SF–2% GF	2	0	0	700	0	280	15	28	784	105	52	0
0% SF–1% GF/1% PPF	1	1		700	0	280	15	28	784	105	26	9.1
0% SF–2% PPF	0	2		700	0	280	15	28	784	105	0	18.2
7.5% SF–2% GF	2	0	7.5	647.5	52.5	280	15	28	769	105	52	0
7.5% SF–1% GF/1% PPF	1	1		647.5	52.5	280	15	28	769	105	26	9.1
7.5% SF–2% PPF	0	2		647.5	52.5	280	15	28	769	105	0	18.2
15% SF–2% GF	2	0	15	595	105	280	15	28	754	105	52	0
15% SF–1% GF/1% PPF	1	1		595	105	280	15	28	754	105	26	9.1
15% SF–2% PPF	0	2		595	105	280	15	28	754	105	0	18.2
reference	0	0	0	700	0	280	15	28	839	105	0	0

In producing fresh geopolymer foams, GBFS, SF, SM and WMP were mixed at low speed for 1 min. Water and WRA were then added to the dry mixture. 1/3 of the water was mixed with WRA and added to the mixture. The mixture formed this way was mixed at low speed for 1 min and at high speed for 2 min. Afterward, foam was added to the mixture, mixing at low speed for 1 min and high speed for 1 min. In the last step, PPF or GF (in hybrid form if available) was added to the mortar mixture and mixed at low speed for 1 min and high speed for 3 min. The geopolymer foams placed in their molds were then sealed in plastic bags. These plastic bags were placed in an oven with a heating rate of 5 °C/min, and the thermal sphere was started immediately. All geopolymer foam samples were cured at 75 °C for 24 h. After heat curing, the specimens were removed from the molds and stored under ambient conditions until the test day.

### 2.3 Experimental Techniques

ASTM C1437 was used to determine the fresh-state characteristics of geopolymer foams. The flow diameters of the mixes were calculated using a flow table. In addition, the densities of the fresh mixtures placed in the molds were measured. Furthermore, physical parameters such as water absorption and apparent porosity were measured on 50 × 50 × 50 mm geopolymer foams using ASTM C642. Another physical parameter, oven-dry density, was assessed on the same samples. After 28 days, the physical properties were determined. The flexural and compressive strengths of geopolymer foams were measured at 7 and 28 days. Mechanical characteristics were determined using prismatic specimens of 40 × 40 × 160 mm. First, a three-point flexural test was performed using the ASTM C348 standard, followed by a compressive strength determination using the C349 standard on the identical specimens.

According to TS EN 1015-18, the amount of water absorbed by geopolymer samples by capillary action was determined. Cube samples of 50 × 50 × 50 mm were created for this purpose. After a 28 day curing period, the samples’ sorption properties were examined. Plate samples of 500 × 500 × 30 mm were constructed to determine the thermal conductivity coefficients of the samples. These specimens were cured for 28 days and dried at 50 °C for 3 days. The thermal conductivity coefficients were then calculated. Thermal conductivity coefficient was determined according to ASTM C518 standard.

The mass loss and mechanical characteristics of geopolymer foams were measured at high temperatures of 200, 400, and 600 °C. Prismatic specimens of 40 × 40 × 160 mm were constructed to determine the high-temperature resistance. First, the specimens’ mass loss was evaluated, followed by their compressive strength after high-temperature exposure. After 28 days of curing, the specimens were subjected to elevated temperatures. The high-temperature effect was achieved using a muffle furnace at a heating rate of 10 °C. After 2 h at the prescribed temperatures, the specimens were taken from the furnace and cooled under laboratory conditions. The samples were treated to 100 freeze–thaw cycles. The specimens’ weight loss and compressive strength were measured following ASTM C666. F-T cycles were carried out on 40 × 40 × 160 mm prism samples, frozen at −20 °C for 7 h and thawed at +4 °C for 5 h. The samples were exposed to F–T cycles for 28 days. The geopolymer foam’s flexural toughness was investigated using prismatic specimens measuring 300 mm long, 60 mm wide, and 25 mm high (25 × 60 × 300). The link between load and midspan deflection was established until the peak load dropped 10% from its starting point. The midspan deflection values were measured using a dial gauge in the three-point load bending test. The dial gauge, placed in the center of the specimen’s span, was immediately attached to the test apparatus and capable of transmitting data to the computer. The three-point bending test used a loading rate of 0.1 mm/min. Figure 3 shows the flowchart of the experimental process in this study.

**Figure 3:**
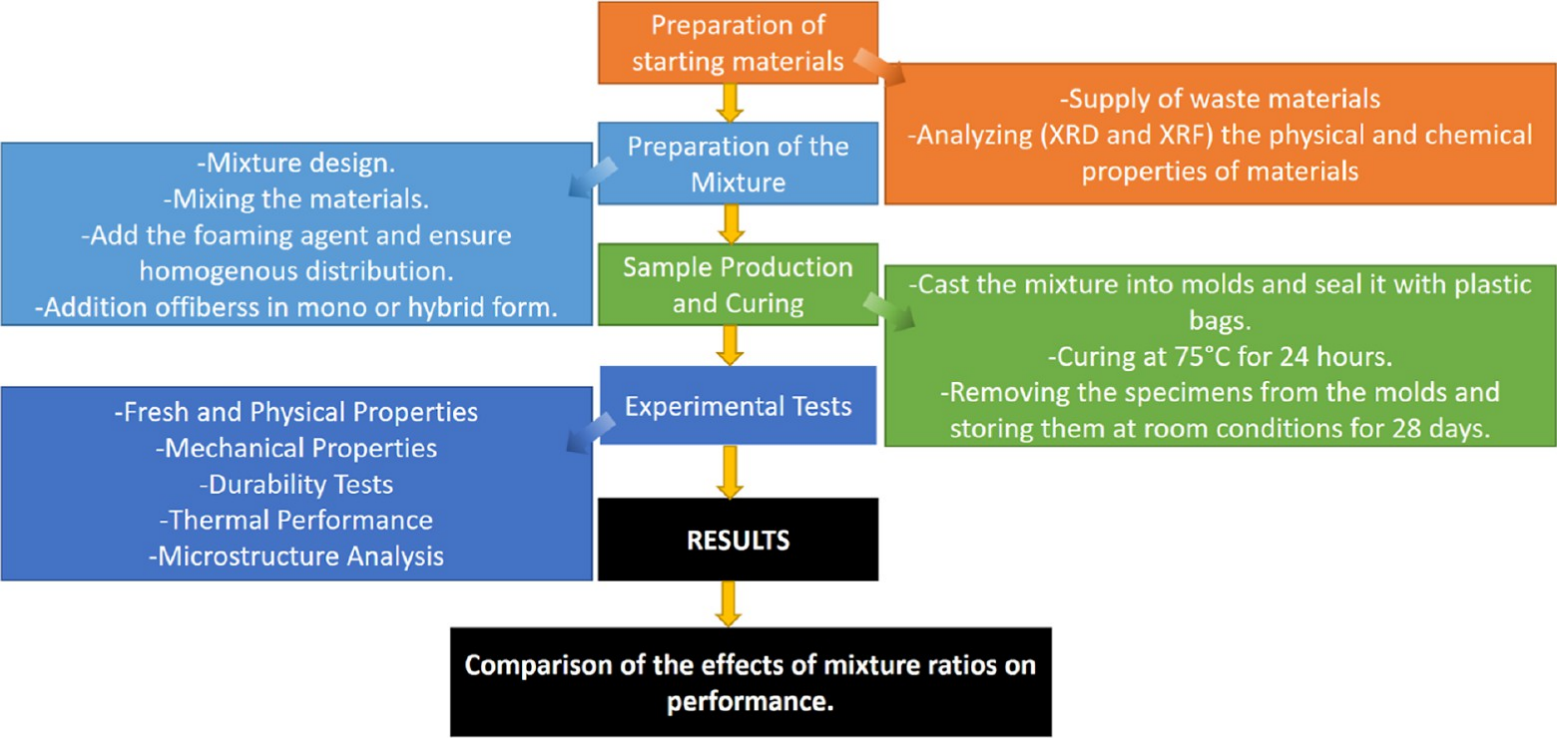
Flowchart of the production process of geopolymer foam concrete.

## 3 Results and Discussion

The Results and Discussion section evaluates the fresh properties (flow diameter), mechanical (compressive strength, flexural strength, load–displacement response), physical (apparent porosity, water absorption and oven-dry density), transport properties (sorptivity), thermal performance (thermal conductivity coefficient), durability performance (frost resistance and high-temperature resistance) and microstructure test results.

### 3.1 Fresh Properties

The flow diameter test results are shown in Figure 4. The flow diameter test was applied to determine the fresh properties of geopolymer concrete. Flow diameter is one of the most critical parameters indicating the workability of fresh concrete. Flow diameter results are essential for evaluating the usability of the data obtained from this experimental study in construction field applications. The absence of SF and fibers in the reference mixture caused the spreading diameter to be 211 mm, higher than all mixtures. This result shows that adding additives such as SF and fibers in the geopolymer mixture reduces the fluidity of the concrete. The addition of SF and fiber increased the viscosity of the concrete, causing internal friction in the internal structure and a decrease in the flow diameter (Figure 4). In mixtures without silica fume, GF created less viscosity than PPF and, therefore, caused a higher spreading diameter. Increasing the SF in the mix to 7.5% and 15% significantly reduced the flow diameter. This is because SF increases the water demand in the mixture due to its fine-grained material. With the increase in SF, more water was absorbed from the mixture (the water that should have been used in the chemical reaction in the mixture was thus absorbed by SF), and the flow diameter also decreased. In addition, the addition of SF to the mixture increased cohesion and reduced fluidity.

**Figure 4:**
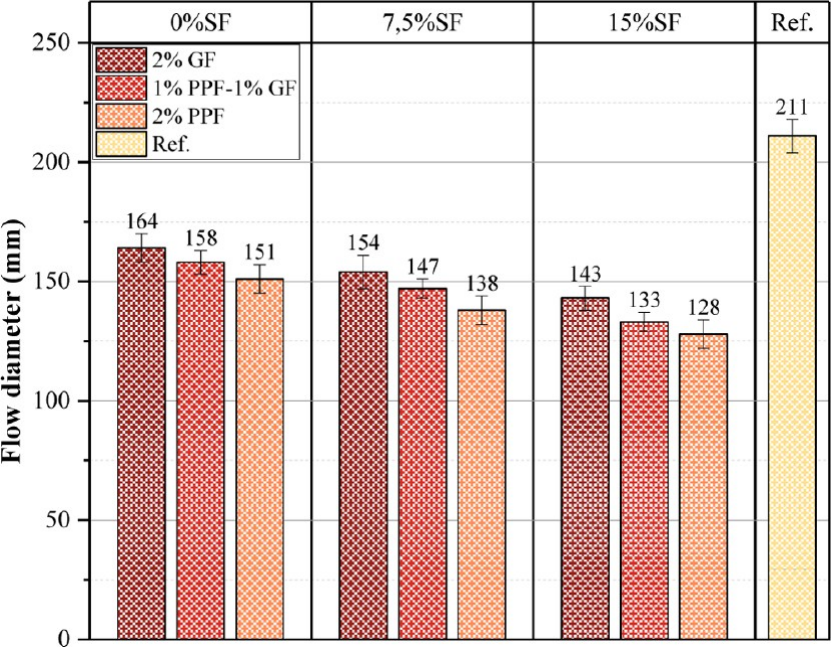
Flow diameter results.

It is seen that the use of GF in the mixture has a more positive effect on the flow diameter compared to PPF. Especially in mixtures containing 0% SF, using 2% GF caused the flow diameter value to be 164 mm. Thus, PPF increased the mixture’s viscosity and caused the spread diameter to be more limited. GF and PPF cause different effects on the fluidity of geopolymer concrete because both fiber types have different effects on the internal structure of the concrete. PPF, has a higher water retention capacity due to its more homogeneous distribution in the matrix. Therefore, it reduced the fluidity of the concrete more than GF. In addition, since the surface area of PPF is greater than GF, it caused more friction and increased viscosity in the matrix. While increasing the ratio of SF in the geopolymer mixture increased the viscosity of the concrete, the type and ratio of fibers reinforced this effect. PPF reduces the workability of the concrete more than GF; therefore, when the SF and fiber ratios are used in large-scale field applications, optimizing these ratios is essential for balanced performance in terms of workability and strength. In their study on the mechanical and workability of GF and PPF additives on slag/fly ash-based geopolymer concrete, Ali et al.^76^ stated that GF has a more positive effect on workability than PPF. However, adding GF and PPF decreased workability compared to the reference sample (without fiber). This study concluded that GF has a more positive effect on workability than PPF. Similarly, it was determined that adding GF and PPF both alone and in a hybrid form decreased workability compared to the reference sample (without fiber).

### 3.2 Physical Properties

The apparent porosity, water absorption and oven-dry density tests were applied to evaluate the physical properties of the tested samples. The results of the physical properties are shown in Figure 5. One of the most critical features of foam geopolymer concrete is its high porosity content, which provides the advantages of low density, high thermal insulation and lightness. The apparent porosity rate of the samples varies between 13.4% and 30.8%. While the 0% SF–1% GF/1% PPF mixture has a low porosity value of 13.5%, the 15% SF–1% GF/1% PPF mix has a high porosity of 30.8% (Figure 4a). Although low porosity generally causes high compressive strength, low porosity typically causes a disadvantage in thermal insulation in foam geopolymer concrete. Using sodium metasilicate (SM) in the foam geopolymer concrete mixture caused the samples to have a tighter structure. Although the porosity was low, especially in the 0% SF–2% GF (23.4%) and 7.5% SF–2% GF (14.2%) samples, the porosity rate of the mixtures was also generally low. This may be due to the use of SM in the mix. Although it was expected that porosity would decrease with the addition of SF to the mixture, using 15% SF in the mix caused an increase in porosity. It is thought that the reason for this situation is that the excess SF in the mixture creates pores in the internal structure. Accordingly, the porous structure in the matrix caused an increase in the water absorption rate in samples containing 15% SF, which caused a decrease in the oven-dry density of samples containing 15% SF. In addition, the addition of GF to the mixtures caused a reduction in porosity. However, due to the fine structure of PPF, it caused porosity to increase because it caused pores instead of filling the voids in the matrix. Chen et al.^77^ reported that adding polypropylene fibers increased the porosity and significantly contributed to the fibers’ compressive strength. In this study, PPF was found to harm the porosity of foam geopolymer concrete.

**Figure 5:**
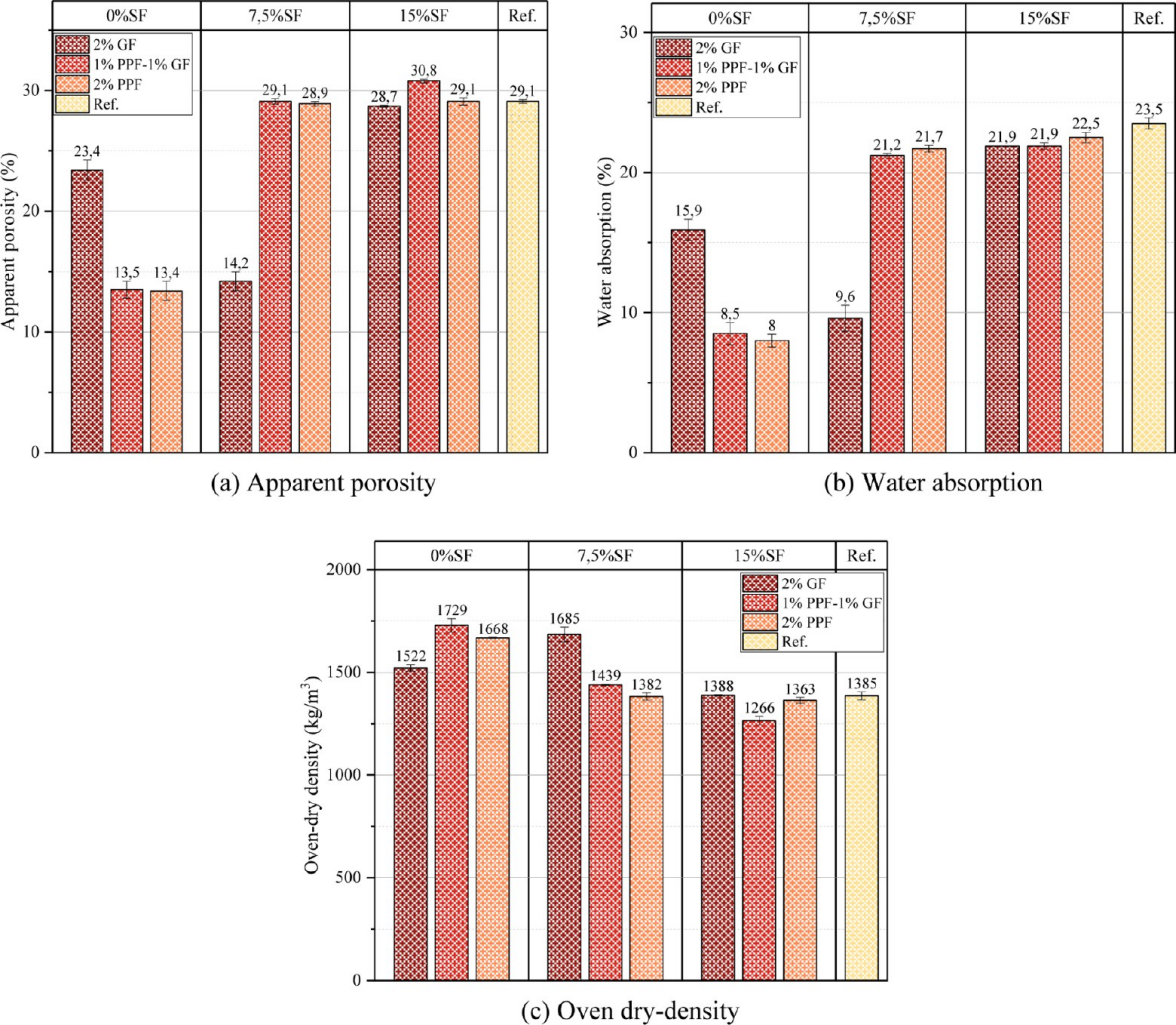
Physical properties of the tested samples (a) apparent porosity (b) water absorption (c) oven-dry density.

Foam concretes generally have a high water absorption rate due to their porous structure and easy water penetration into the concrete. The water absorption rate of the samples varies between 8% and 23.5% (Figure 4b). While 0% SF–2% PPF (8%) has the lowest water absorption rate, the reference mixture has the highest of 23.5%. Therefore, foam concrete’s low water absorption rate has resulted in a more durable structure. In addition, in the case of using SM in the mixture, SM has increased the permeability of the concrete by controlling water absorption. Since GF is generally effective in preventing the water absorption rate, the 7.5% SF–2% GF mix has a low water absorption rate of 9.6%. However, the increase in the water absorption rate up to 21.9% in the 15% SF–2% GF mixture caused the positive effect of using SF at high doses on the water absorption rate of GF to decrease. PPF was not as effective on the water absorption rate as GF. The water absorption rates of mixtures containing PPF are generally high, with the lowest value being 0% SF–2% PPF (8%). However, as the SF rate increased (7.5% and 15%), the water absorption rates of mixtures containing PPF also increased, reaching 22.5%. This shows that PPF can increase the pore structure and allow water passage, mainly when used with high SF rates.

Another essential feature of foam concrete is its low density. Since foam concrete has a light structure, it is used in construction site applications. The densities of the samples are generally within the acceptable range (1266–1729 kg/m^3^) for foam concrete (Figure 4c). In addition, using SM in the mixture caused the concrete to have a tighter bond structure, which increased the density. In line with the values obtained from Figure 5, using waste materials (marble powder), recyclable binders (GBFS and SF) and SM in foam geopolymer concrete has demonstrated an environmentally friendly approach. Moreover, using SM, porosity is controlled, and the performance of geopolymer foam concrete is improved.

### 3.3 Mechanical Properties

#### 3.3.1 Compressive Strength

The 7 day and 28 day compressive strength results are presented in Figure 6. It aimed to investigate the effects of waste materials such as WMP, GBFS SM, and hybrid fibers (GF and PPF) on the mechanical performance of foam geopolymer concrete using compressive strength, which results in an environmentally friendly concrete type. For this reason, the samples’ 7- and 28 day compressive strengths were presented. Silica fume is an industrial byproduct with a high amorphous silicon dioxide (SiO_2_) content. It is often used to increase the strength of concrete. However, several possible reasons exist why silica fume may reduce the compressive strength of geopolymer foam concretes. Since silica fume contains high-purity amorphous silica, it reacts rapidly with sodium metasilicate to form a large amount of soluble silicate at an early age. This adversely affects the polymerization process and prevents homogeneous curing of the matrix. Sodium metasilicate provides high alkalinity, which increases the solubility of silica fume. However, excess silica solubility can cause precipitates by disturbing the pH balance of the mixture. These residues form weak spots in the geopolymer matrix and weaken the integrity of the material. The fast and intense reactions between sodium metasilicate and silica fume can make it challenging to obtain a homogeneous mixture. This can lead to phase separation and the formation of heterogeneous structures. Phase separation reduces the material’s mechanical strength and its long-term durability. However, the reactions between sodium metasilicate and silica fume can be exothermic (heat releasing). Excessive heat release can increase the internal temperature of the mixture, leading to drying shrinkage cracks. These cracks increase porosity and reduce compressive strength. Furthermore, silica fumes contain very fine particles with a large surface area. These fine particles may agglomerate in the mixture, preventing their uniform distribution in the matrix and leading to the formation of voids. These voids, formed as a result of agglomeration, increase porosity.

**Figure 6:**
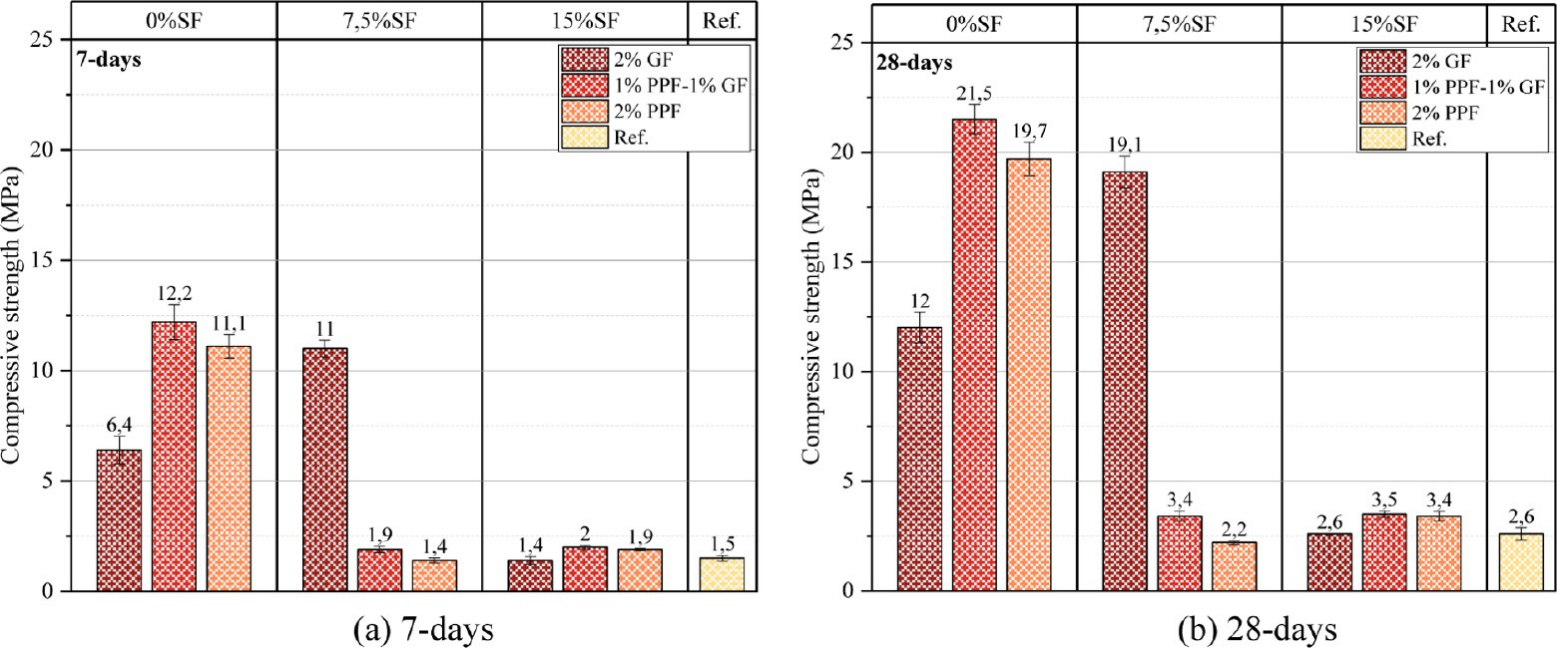
Compressive strength of samples (a) 7 (b) 28 day.

It is seen that although the 0% SF–2% GF mixture reached a compressive strength of 6.4 MPa in 7 days, it reached a compressive strength of 12 MPa in 28 days. This result shows that although the foam geopolymer concrete containing slag and GF has a relatively low early age strength, it increases with time. The 7 day and 28 day compressive strengths of the 0% SF–1% GF–1% PPF mixture are 12.2 and 21.5 MPa, respectively. Thus, it shows the positive effect of adding PPF to the mix on both early and long-term compressive strength. However, using SF in foam geopolymer concrete hurts the compressive strength. For example, the 7 day and 28 day compressive strengths of the 7.5% SF–2% GF mixture are 1.9 and 3.4 MPa, respectively, indicating the negative effect of SF on compressive strength. The reason for this situation is that if SF is used excessively, it negatively affects the geopolymerization process in the matrix and causes voids, which causes the compressive strength to decrease.

The excessive amount of SF in the mixture does not react in the geopolymerization process and prevents the sufficient formation of the bond structure (C–S–H and A–S–H) that strengthens the matrix. Therefore, the optimum use of the SF ratio in foam geopolymer concretes is very important regarding compressive strength. Strong phases cannot be formed if the SF ratio is not used at the optimum level due to the insufficient reaction of alkali activators with binders. Adding GF and PPF has increased the compressive strength due to the control of crack formation and the increase in energy absorption. In particular, the hybrid of GF and PPF has positively affected compressive strength. This situation is because GF distributes the stresses in the matrix by forming a network structure, and PPF prevents micro cracks. The synergistic effect of combining these two different fiber types increased the compressive strength. Even in the mixtures without SF, the impact of the combination of these two fiber types is more pronounced. An important point is that when these fiber combinations are used together with SF, they harm the compressive strength, and the effect of the fibers on the compressive strength is limited. Sabu and Karthi^78^ reported that adding PPF increases compressive strength. However, Ali et al.^76^ reported that adding GF and PPF decreased the compressive strength. In this study, adding GF and PPF positively affected the compressive strength. Moreover, Kantarcı^79^ reported that including GF fiber enhanced the compressive strength thanks to geopolymer gel covering the fiber surface and the strong adhesion between GF and geopolymer matrix. However, Kantarcı^79^ indicated the negative effect of PPF on compressive strength. Nanditha et al.^80^ also emphasized that the additional GF increased the compressive strength of geopolymer concrete.

The comparison of apparent porosity, oven-dry density and compressive strength results are shown in Figure 7. Apparent porosity shows the pore structure of the samples and the amount of voids in the matrix accordingly. Oven-dry density represents the mass-volume relationship of the samples. As seen in Figure 7, as the apparent porosity of the samples increases, oven-dry density also decreases significantly. The increase in voids in the matrix causes the pores to increase, and these pores also cause the mass to reduce and, thus, the density to decrease (*R* ^2^ = 0.94 relationship). Figure 7 shows that the increase in the ratio of SF in the mixture causes the formation of reactive particles that do not form sufficient bonds in the matrix, and in this case, the pore number increases, causing the porosity to increase and the density to decrease. GF and PPF additives cause the density to increase by reducing the pore number in the matrix.

**Figure 7:**
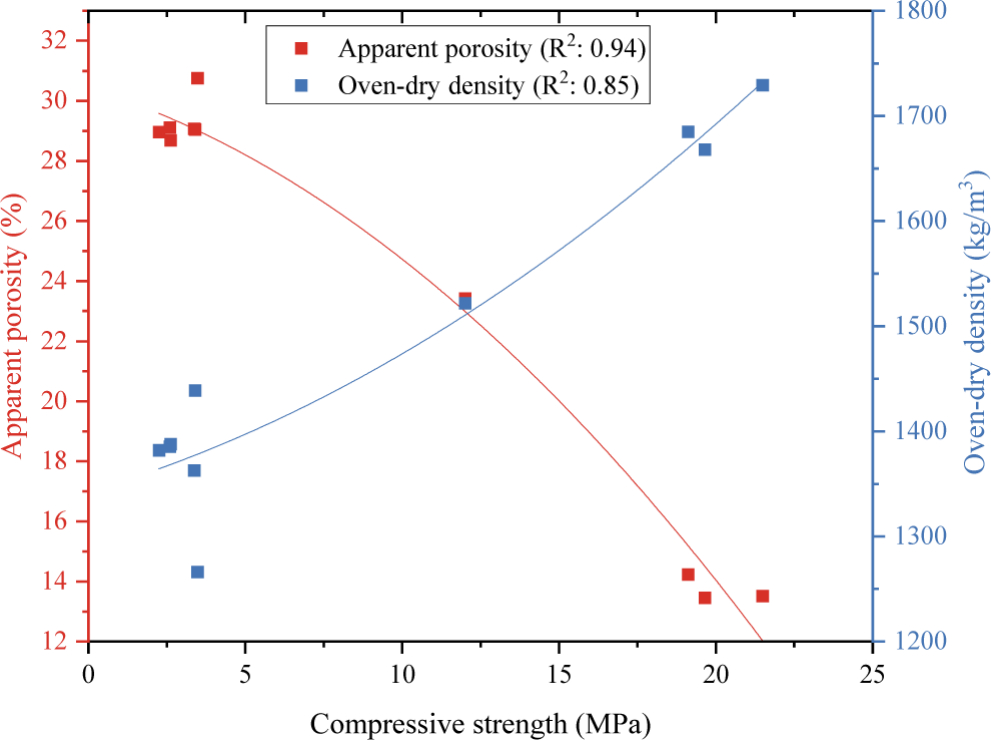
Comparison of apparent porosity, oven-dry density and compressive strength.

It is seen in Figure 7 that there is a strong relationship (*R* ^2^ = 0.85) between oven-dry density and compressive strength. The increase in the density of the samples also increased the compressive strength. Since denser samples have a tighter internal structure, their compressive strength is also high. The decrease in the voids in the matrix caused the stresses to be distributed more homogeneously. The homogeneous distribution of the stresses also caused the compressive strength to increase (for example, the high compressive strength of the 0% SF–1% GF/1% PPF sample). Similarly, the use of fibers in the mixtures increased the density and, therefore, caused the compressive strength to rise.

There is an inverse relationship between apparent porosity and compressive strength. The increase in pores in the matrix caused stresses to accumulate between these voids, allowing microcracks to form more easily. For this reason, the compressive strength decreased. The increase in the SF ratio in the mixture and the decrease in the fiber ratio increased apparent porosity and caused the compressive strength to decrease.

#### 3.3.2 Flexural Strength

The 7 and 28 day flexural strength results are presented in Figure 8. Among the samples without SF, the highest values were reached in the 0% SF–1% GF/1% PPF sample regarding both early and late strength. This shows that when SF is not used in foam geopolymer concrete, the hybrid of GF and PPF additives increases the binding capacity in the matrix and positively affects flexural strength. The absence of SF in the matrix shows that the slag and fibers are bonded more homogeneously and effectively. SF in foam geopolymer concrete has negatively affected flexural and compressive strength. The flexural strengths of the samples using SF have generally decreased. For example, the 7 and 28 day flexural strength results of the 7.5% SF–2% PPF sample are 0.8 and 1.4 MPa, respectively. These results are the lowest among the samples. This is because when SF is used excessively in the matrix of foam geopolymer concrete, the fiber–matrix interaction is negatively affected and weakened. The excess SF, which does not undergo a geopolymerization reaction in the matrix, makes the matrix denser and limits the contribution of the fibers to the mechanical performance. Therefore, it has also reduced the influential role of the fibers in resisting the stresses in the samples under flexural loading. The 28 day compressive strength of the reference mixture without SF and fibers is 2.2 MPa, which is higher than the compressive strength of some SF and fiber-added samples.

**Figure 8:**
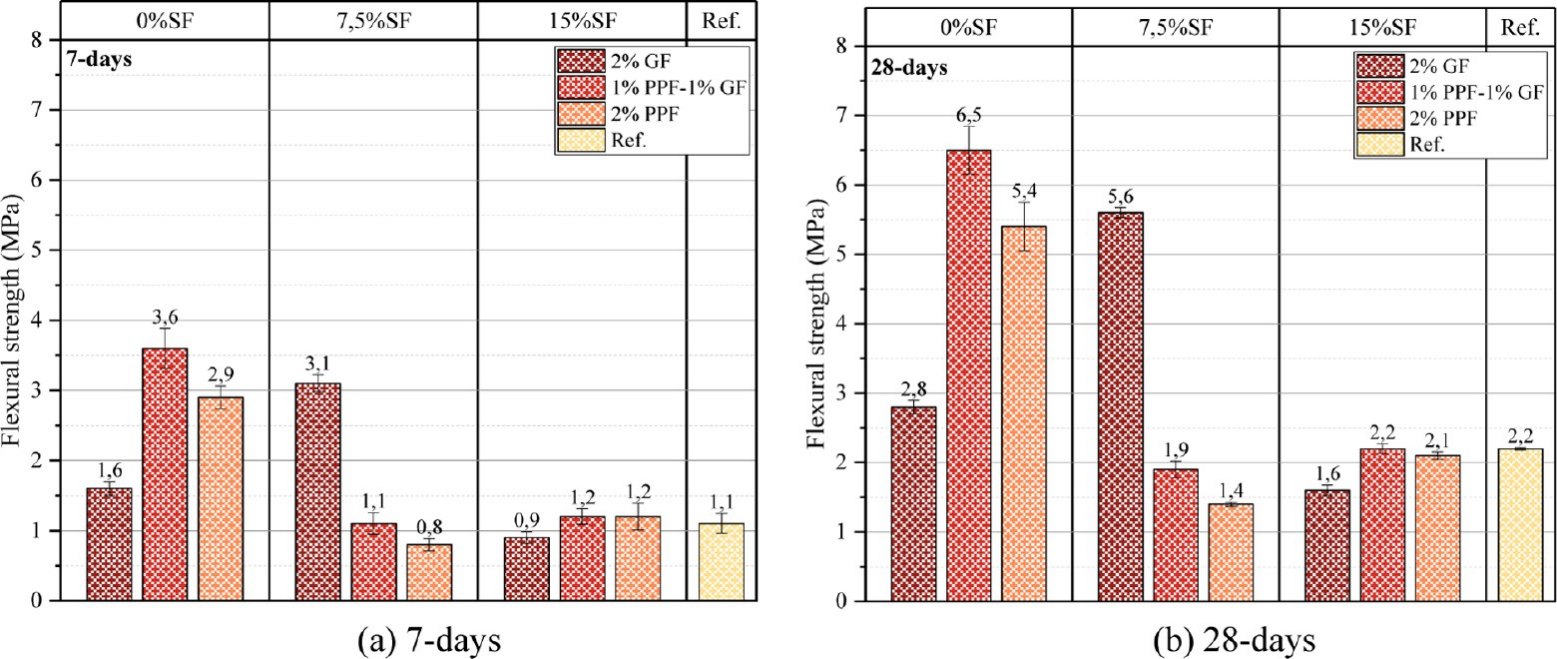
Flexural strength of samples (a) 7 (b) 28 day.

Using GF and PPF fibers together was more effective in improving flexural strength performance. The 7 and 28 day flexural strengths of the 0% SF–1% GF/1% PPF sample were the highest results, with 3.6 and 6.5 MPa, respectively. Both fiber types showed a more effective crack bridging effect in samples under bending moment. Due to this feature of the fibers, crack formation and propagation were controlled, increasing the flexural strength. The impact of using the fibers alone on the flexural strength is different. For example, although GF fibers had a positive effect on the flexural strength in the 0% SF–2% GF mixture (28 day strength 2.8 MPa), a higher flexural strength (5.4 MPa) was obtained in the 0% SF–2% PPF mixture where PPF was used at a rate of 2%. This shows that PPF is more effective in flexural strength than GF.

The use of SM in foam geopolymer concrete caused the chemical reaction to accelerate and the strength of the matrix to increase. SM, GBFS and SF reacted with alkali activators and accelerated the formation of SiO_4_ and AlO_4_ structures. For this reason, the 7 day early flexural strengths of the samples reached high values. For example, the 7 day flexural strength of the 0% SF–1% GF/1% PPF sample gave a very high result of 3.6 MPa (Figure 7a). The effectiveness of SM also changed depending on the SF. Although the flexural strength was low in samples with high SF content (for example, 15% SF–2% GF, 15% SF–1% GF/1% PPF), the flexural strength of samples with low SF content (for example, 0% SF–1% GF/1% PPF) was higher. In the 0% SF–1% GF/1% PPF sample, it can be said that SM supports the ability of the fibers to prevent crack propagation by increasing the activation of the slag. SM generally caused an increase in density in foam geopolymer concrete. However, using SM and SF increased the matrix’s density considerably. In this case, it caused both the formation of pores in the matrix and the decrease in the effectiveness of the fibers. Ali et al.^76^ reported that additional PPF decreased the flexural strength of slag/fly ash-based geopolymer concrete, in which GF had positively affected the flexural strength.

It should be noted that the PPF resulted in increased flexural strength of foam geopolymer concrete, although additional PPF decreased the flexural strength of slag/fly ash geopolymer concrete. Hussein et al.^81^ found that mixing GF with nanosilica lowered flexural and compressive strengths when subjected to sulfuric acid, magnesium sulfate, and sodium chloride. According to Hussein et al.,^81^ the reason for the decrease in the flexural strength of the samples under the effect of acid and sulfate is that the resistance to external factors such as acid and sulfate decreases due to the porous structure of GF and nanosilica. However, in this study, it was determined that the use of GF in foam geopolymer concrete reduces the apparent porosity value. Therefore, it should be noted that the effect of using GF in terms of flexural strength and permeability differs from that of using nanosilica or foam concrete.

#### 3.3.3 Flexural Load–Displacement Relationship

Figure 9a shows the flexural load–displacement relationship of FS-free geopolymer foams. The mix using 2% FF exhibited a satisfactory bearing capacity regarding peak load. This can be explained by the glass fibers’ high tensile strength (3450 MPa) and stiffness (72 GPa elastic modulus). Although the brittle nature of the glass fibers limits the energy absorption capacity after fracture, it effectively carries the stress accumulation up to the peak load point. Since polypropylene fibers have a high elongation capacity (25%), they support energy absorption in the larger displacement range in the graph. In the hybrid blend, the peak load value remained high due to the contribution of glass fibers.

**Figure 9:**
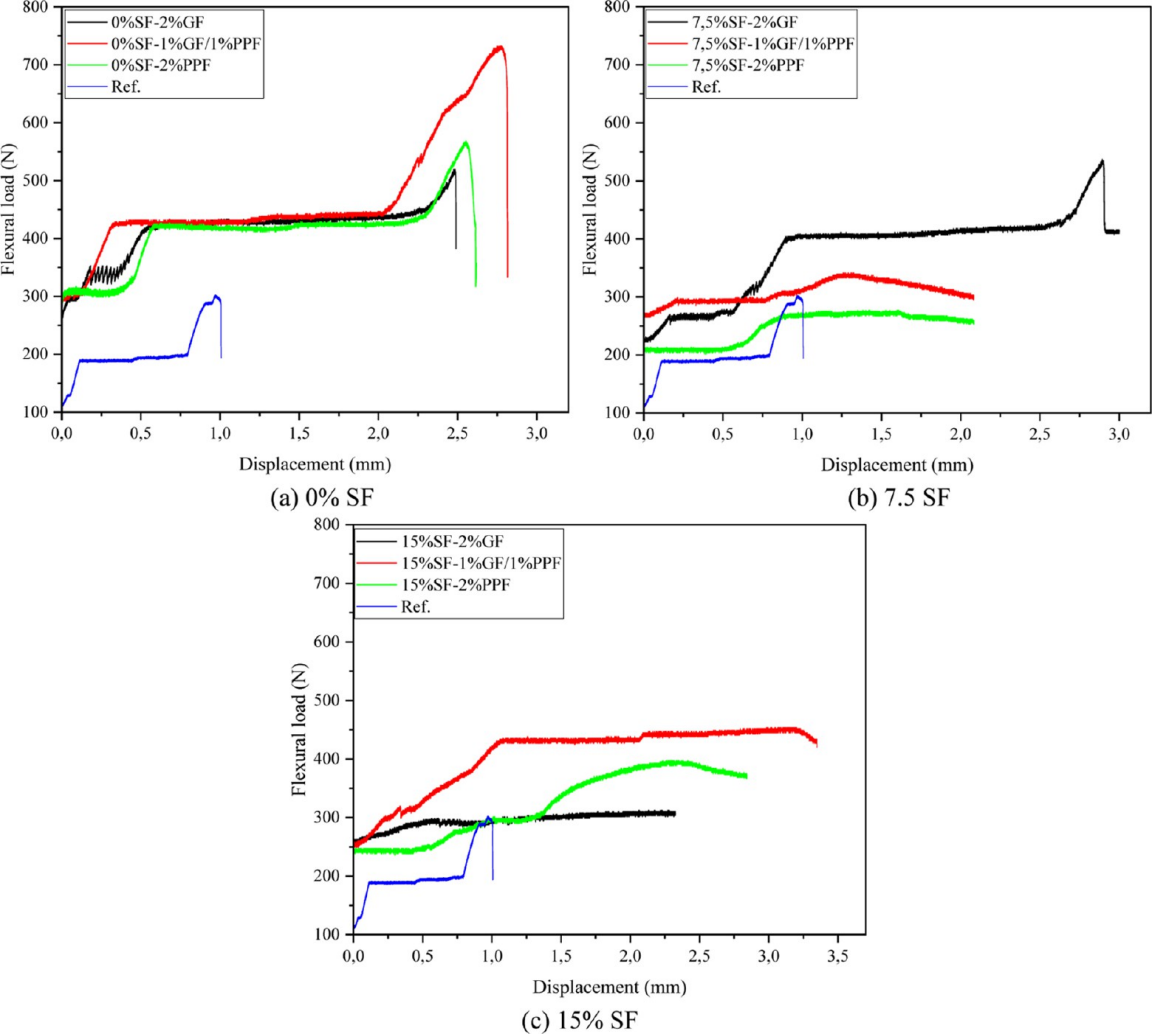
Load–displacement curves of geopolymer foams.

The PPF, on the other hand, has an elongation property that permits energy absorption once a fracture occurs. By working synergistically, this combination prevented fracture propagation and improved the overall performance. The improved performance of the hybrid fibers was due to their capacity to suppress microcrack propagation.^23^ The flexural strength data in Section 3.3.2 corroborate this. For example, the 7 and 28 day flexural strength for the 0% SF + 1% GF + 1% PPF blend was measured as 3.6 and 6.5 MPa, respectively, highlighting the superiority of the hybrid fibers. The peak load values were lower in the reference mix without fiber additives. This can be attributed to the low density (1266 kg/m^3^) and high porosity (30.8%) values in Figure 5c. The addition of fibers strengthened the bonding within the matrix and increased the density (e.g., density 1729 kg/m^3^ in the hybrid mix), leading to better mechanical performance. As a result, it is recommended that both the strength and deformation capacity of geopolymer foams containing 0% SF be increased by optimizing hybrid fiber ratios.

As can be seen in Figure 9b, when the SF content is 7.5%, a change in both load-carrying capacity and displacement capacity is observed. Different fiber types (GF and PPF) and hybrid combinations affected the mechanical properties of the matrix to various degrees. The mixture with 2% GF reached the highest bearing capacity (about 550 N). This can be attributed to the glass fibers’ high tensile strength and stiffness. Displacement: The displacement value under maximum load was more than 2.50 mm. The brittle nature of the glass fibers limited the postfracture deformation but increased the load-carrying capacity. This blend, in which PPF is used entirely, has a lower bearing capacity (approximately 275 N). The displacement at the maximum load point is lower than that of glass fibers at 1.35 mm. The flexible structure of PPF fibers can explain this, but insufficient efficiency could not be obtained due to inadequate adherence. Hybrid fibers optimized the bearing capacity (approximately 350 N). The stiffness of the glass fibers is effectively combined with the energy absorption of the PPF. SF increased the matrix density in the specimens containing 7.5% SF and strengthened the bonding (for 2% GF). This favorably affected the load-carrying capacity. However, it was determined that excessive amounts of SF may increase porosity (Figure 5a, 14.2% porosity) and weaken fiber–matrix interaction. In the blend with hybrid fibers (1% GF + 1% PPF), peak load and displacement values were moderate. This indicates that hybrid fibers prevent crack formation and support energy absorption.

Glass fibers contributed to the load-carrying capacity by increasing the stiffness of the matrix (Figure 9c). However, the high SF content (15%) limited the bonding efficiency and crack bridging capacity, leading to low deformation capacity. Hybrid fibers slowed down the crack propagation and increased the deformation capacity with a synergistic effect. While glass fibers increased the load-carrying capacity, PPF fibers performed more balancedly by providing energy absorption. The flexible structure of polypropylene fibers activated the crack-bridging mechanism and improved the deformation capacity. However, due to the low tensile strength, the load-carrying capacity remained lower than that of hybrid fibers. In Figure 9c, the synergistic effect of hybrid fibers was determined. While glass fibers increased the load-carrying capacity, PPF fibers contributed to the deformation capacity. This balance resulted in the highest peak load and balanced deformation performance.

Also, in the flexural load–displacement curves in Figure 9, a “plateau” region is observed where the displacement value continues to increase while the load remains constant within a specific range. In geopolymer foams, cracks begin to form in the microstructure (e.g., within the binder matrix or at fiber–matrix interfaces) during initial flexural loading. Even if the cracks propagate rapidly at a certain point, the presence of fibers ensures stable crack propagation. The total load level can remain constant in fiber-reinforced materials as the load-carrying capacity along the microcracks is shared between the fibers and the matrix. The increase in displacement during this process is due to the absorption of energy by the fibers. The load-carrying capacity remains constant, especially for 2% GF and 1% GF-1% PPF (e.g., Figure 9a), indicating that the fibers stabilize the stress by bridging matrix cracking. The fibers slow the propagation of cracks in the matrix due to their elastic and plastic deformation capacity before rupture. The increase in displacement in the plateau region indicates the load-carrying and energy absorption capacity of these fibers at the crack tips. The plateau region can be particularly pronounced in composites with weak matrix–fiber interfaces. The interface may exhibit sliding behavior under load, and while this sliding behavior may cause the load to remain constant, deformation may continue. This effect can be more pronounced in low-stiffness polypropylene fibers (2% PPF) because the flexibility of these fibers increases the energy absorption capacity. The plateau region we see is the limit of plasticity of the material. If you keep the load steady, the enlargement in strain suggests that the binder section and fibers can absorb energy without delay. Examples include 1% GF–1% PPF hybrid fiber blends, where crack propagation becomes more efficient and the load-carrying capacity remains unchanged.

In addition, pores in the microstructure of geopolymer foams can also enable crack propagation speed reduction. This is expected because porous structures are energy-dissipative materials, so the material deforms closer to yield when carrying a load. This relates to keeping the plateau zone lasting. These findings indicate that tuning fiber–matrix interactions can improve the deformation ability of materials. This plateau behavior is essential data for the structures’ damage tolerance and energy absorption capacity.

The 2% GF blend without SF reached the highest peak load value of 733 N (Figure 10a). This is due to the high stiffness of the glass fibers and their strong bonding properties with the matrix. Glass fibers maximized the load-carrying capacity by increasing the crack bridging effect. The hybrid fiber blend (1% GF + 1% PPF) reached a value of 569 N and exhibited a lower performance than the glass fiber blend. This is attributed to the flexible nature of PPF, which offers a lower load-carrying capacity. The blend with 2% PPF fibers performed slightly lower than the hybrid fiber blend with 542 N. The capacity of PPF fibers to slow crack propagation is more limited. Therefore, it lacks the high stiffness provided by the glass fiber additive. When the SF content was increased to 7.5%, the peak load value of the blends decreased compared to 0% SF. This decrease is due to the limitation of the bond strength of SF due to the increase in porosity. Although the blends with fiber additives increased the load-carrying capacity compared to the reference blend, the peak load values of the blends with 7.5% SF were generally lower than those of 0% SF. The 2% GF blend provided the highest peak load of 535 N. SF, which weakened the fiber–matrix interaction and limited the crack bridging capacity. The hybrid fiber blend (1% GF + 1% PPF) performed lower than the GF blend but higher than the reference blend with a peak load of 342cN. The 2% PPF blend exhibited the lowest peak load value, reaching only 277 N. This is related to the low tensile strength of PPF and the weakening of bonding by SF by increasing porosity. In geopolymer foams with 15% SF and 2% GF, the lowest peak load value of 299 N was obtained. This result shows that the increase in SF limits the performance of glass fibers. The hybrid fiber blend (1% GF + 1% PPF) showed higher peak performance than the 2% GF blend, reaching 445 N. The hybrid fibers increased the crack bridging capacity by increasing the load-carrying capacity. The blend with 2% PPF showed a value close to the reference blend with 398 N. This indicates that PPF could not contribute sufficiently due to its bond weakness at a high SF ratio. Fiber additives, especially glass fiber (2% GF), generally increased the peak load values. This is associated with the ability of the fibers to limit crack propagation and increase the load-carrying capacity. The PPF fiber additive, although contributing to the deformation capacity, increased the load-carrying capacity to a limited extent due to its low tensile strength. Increasing SF content (0% → 7.5% → 15%) led to an overall decrease in peak load values. This is related to the high porosity and matrix-bonding weakness of SF. The hybrid fibers (1% GF + 1% PPF) showed a balanced performance due to their crack bridging and energy absorption mechanisms. However, it presented lower peak load values than the GF blend due to the effect of doping ratios or SF. As a result, a 2% GF blend is recommended for applications where load-carrying capacity is of primary importance. Hybrid fiber additives can be considered if deformation capacity and energy absorption are essential.

**Figure 10:**
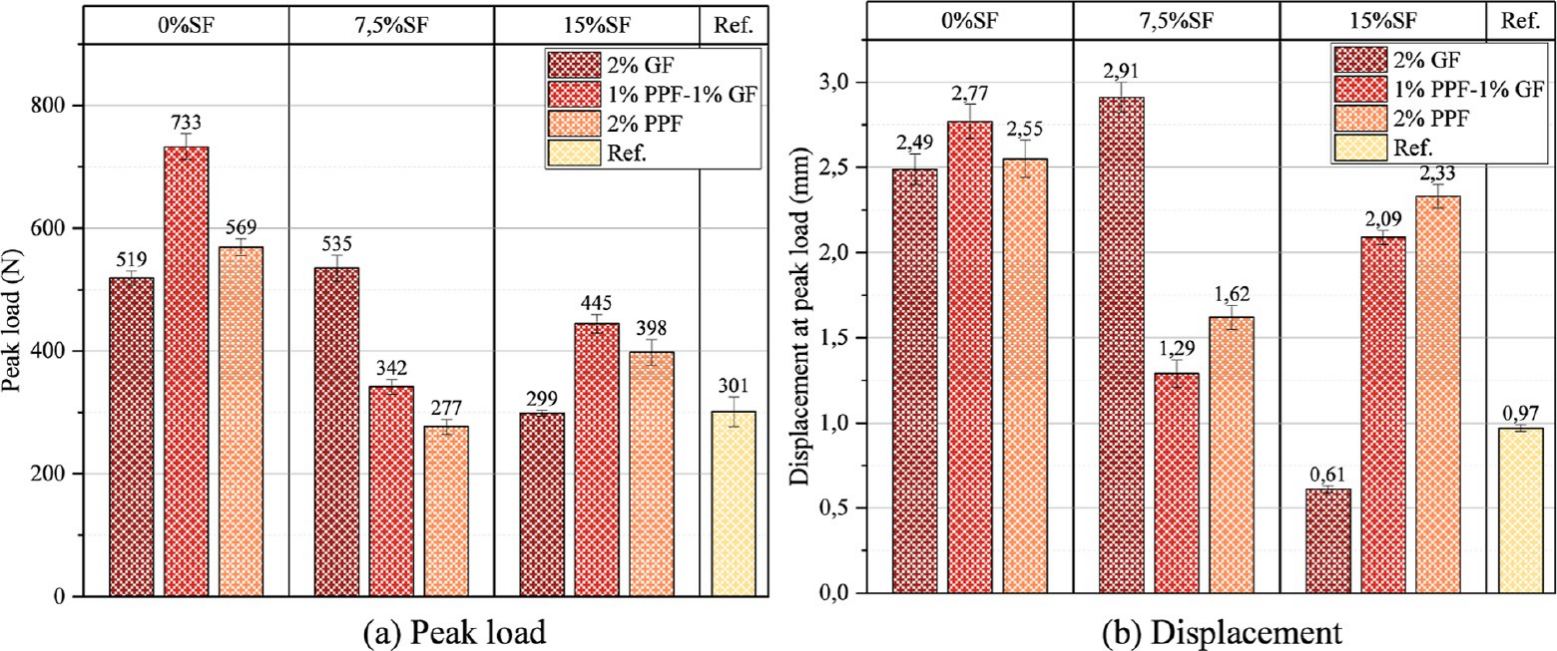
Peak load and displacement behavior of geopolymer foams.

Figure 10b shows the displacement values of geopolymer foams at peak load. In geopolymer foams using 0% SF, the change in fiber type did not affect the displacement value much. It was determined that the synergistic effect of hybrid fibers increased the deformation capacity and improved the flexural strength. It was also determined that the energy absorption capacity was increased with the high flexibility of PPF. When 7.5% SF was used, the displacement value of the hybrid fiber blends decreased significantly (about two times). This may be due to the incompatibility at the fiber–matrix interface. Also, the same blend in 2 w % of PPF, even with fibers, increased energy absorption, but deformation capacity was partially lost adverse due to the binder. The displacement at peak load was very low for 2% GF in mixtures with 15% SF. Then, the low-strain failure model was inappropriate in explaining the measured fiber stiffness since too much of this deformation capacity resided in a matrix with excessive strain. As the SF ratio increases, a more significant portion of the binder phase becomes hardened, resulting in lower deformation capacity. All fiber-reinforced mixtures with 15% SF are presented to have considerably lower displacement values.

In addition, since the use of 15% SF created significant differences in geopolymerization, the displacement values decreased. GF (2% GF) increases the deformation capacity by forming a better bond with the binder. PPF provides a more flexible structure, but the increase in the SF ratio limits the deformation capacity of both fibers. The hybrid fibers (1% GF–1% PPF) had higher deformation in the blends with 0% SF, while those containing at least 15% did not act synergically. This is due to the binder phase’s brittle nature and the fiber–matrix interface mismatch. In addition, geopolymerization reactions are also an essential factor in this process, considering that they are effective between the fiber–matrix interface. The deformation capacities in the graph show a decreasing trend with an increasing SF ratio. Fiber additives exhibit different effects depending on the chemical structure and stiffness of the binder phase.

### 3.4 Transport Properties

The sorptivity results of the samples are presented in Figure 11. The sorptivity test was applied to determine the water absorption capacity of foam geopolymer concrete and the rate of water diffusion into the matrix. In light of the results obtained from this test, evaluating the relationship between water absorption leading to deterioration and cracking of concrete and strength is crucial. Both GF and PPF fibers have a significant effect on the water absorption capacity. Using both GF and PPF in samples containing 0% SF and 7.5% SF decreased the water absorption rate. This situation is because both GF and PPF additives prevent water from entering the matrix and reduce the permeability of the samples. The increase in the SF ratio in the mixture also caused the permeability to increase. As emphasized above, the sorptivity value also increases due to increased porosity when SF is added to the mixture. Since excessive amounts of SF cause pores in the matrix, water absorption occurs in these pores. This also caused the water absorption capacity of the samples to increase. In addition, the hybridization of GF and PPF decreased the sorptivity value. GF and PPF fibers filled the microvoids in the matrix and reduced the water absorption capacity. Moreover, the hydrophobic structure of PP fibers also caused the water absorption capacity of the samples to decrease.

**Figure 11:**
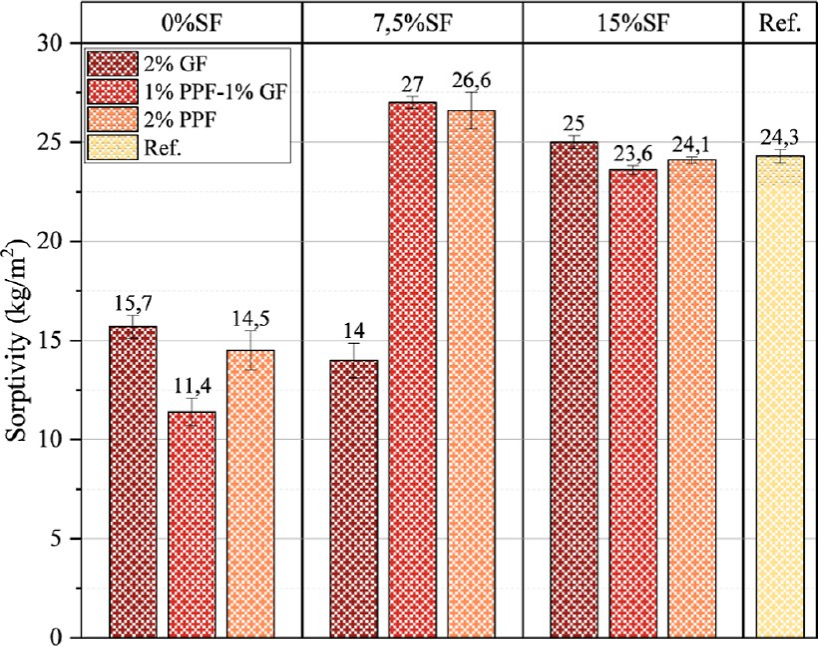
Sorptivity results of tested samples.

### 3.5 Thermal Performance

Thermal conductivity coefficient results are presented in Figure 12. A Thermal conductivity coefficient test was applied to determine the heat conduction capacity of foam geopolymer concrete. The results in Figure 12 are essential for evaluating foam geopolymer concrete’s energy efficiency and resistance to temperature changes. The thermal conductivity capacity of samples without SF is higher than that of others. Thermal conductivity capacity decreased with the addition of SF to the mixture. In addition, GF and PPF fibers have distinctly different effects on thermal conductivity. For example, while the thermal conductivity value of the mixture containing 0% SF and 2% PPF is 1.066 W/m.K, the conductivity value decreases to 0.823 W/m.K in the same mix without SF when there is only 2% GF. This shows that glass fibers (GF) offer lower thermal conductivity than polypropylene fibers (PPF). It is thought that this situation is caused by the more flexible and lightweight structure of PPF fibers. When GF and PPF fibers were used separately, they caused different effects on thermal conductivity. For example, the thermal conductivity value of the mixture of 7.5% SF and 1% GF/1% PPF was measured as 0.806 W/m K, which is lower than the mixture containing only 2% GF (1.061 W/m K). This result shows that glass fibers may be more disadvantageous in heat conduction when used alone, but when combined with PPF, thermal conductivity is significantly reduced. It is also thought that using WMP in foam geopolymer concrete causes a decrease in thermal conductivity. It is also believed that WMP increases the insulation capacity of the samples by shortening the heat conduction networks in the matrix due to its small particles. For these reasons, it is seen that the use of waste materials such as WMP in foam geopolymer concrete has a positive effect in terms of conductivity.

**Figure 12:**
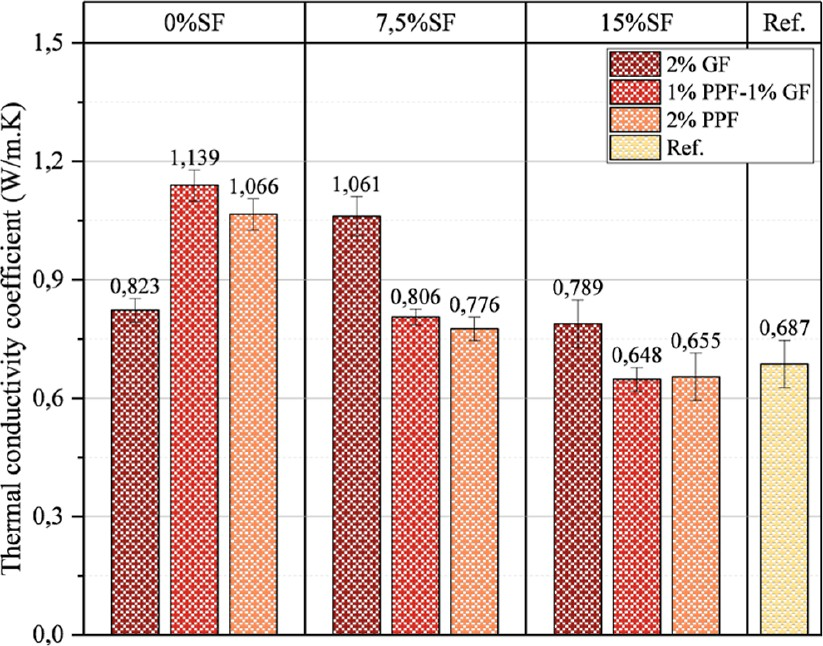
Thermal conductivity coefficient results of tested samples.

### 3.6 Durability Performance

#### 3.6.1 Frost Resistance

The samples’ compressive strengths and mass losses after the freeze–thaw test are presented in Figure 13. The compressive strength losses of the samples without SF are less than those containing SF. For example, the 28 day compressive strength of the mixture containing 0% SF and 1% GF/1% PPF is 21.5 MPa, and the compressive strength after freeze–thaw is 18.8 MPa (Figure 13a). In contrast, these values are quite low in the mixtures containing 15% SF; the 28 day compressive strength of the mixture containing 15% SF and 2% GF is only 2.6 and 2.2 MPa after freeze–thaw. These results show that increasing the amount of SF in the mixture causes a decrease in the freeze–thaw resistance. These results also show that the excess amount of SF in the matrix causes a porous structure, which also causes a reduction in the freeze–thaw resistance. The increase and decrease in volume during the freezing and thawing of water around the pores during freeze–thaw cycles caused extra stress. These stresses caused decreases in compressive strength. Hybridization of fibers caused an increase in freeze–thaw resistance in mixtures not containing SF. PPF, in particular, can help the material maintain its strength during freeze–thaw cycles by preventing crack expansion at low temperatures. The increase in SF in foam geopolymer concrete also significantly reduced the freeze–thaw resistance. After the freeze–thaw test of the 7.5 SF %2 PPF sample, the compressive strength decreased to 2.1 MPa.

**Figure 13:**
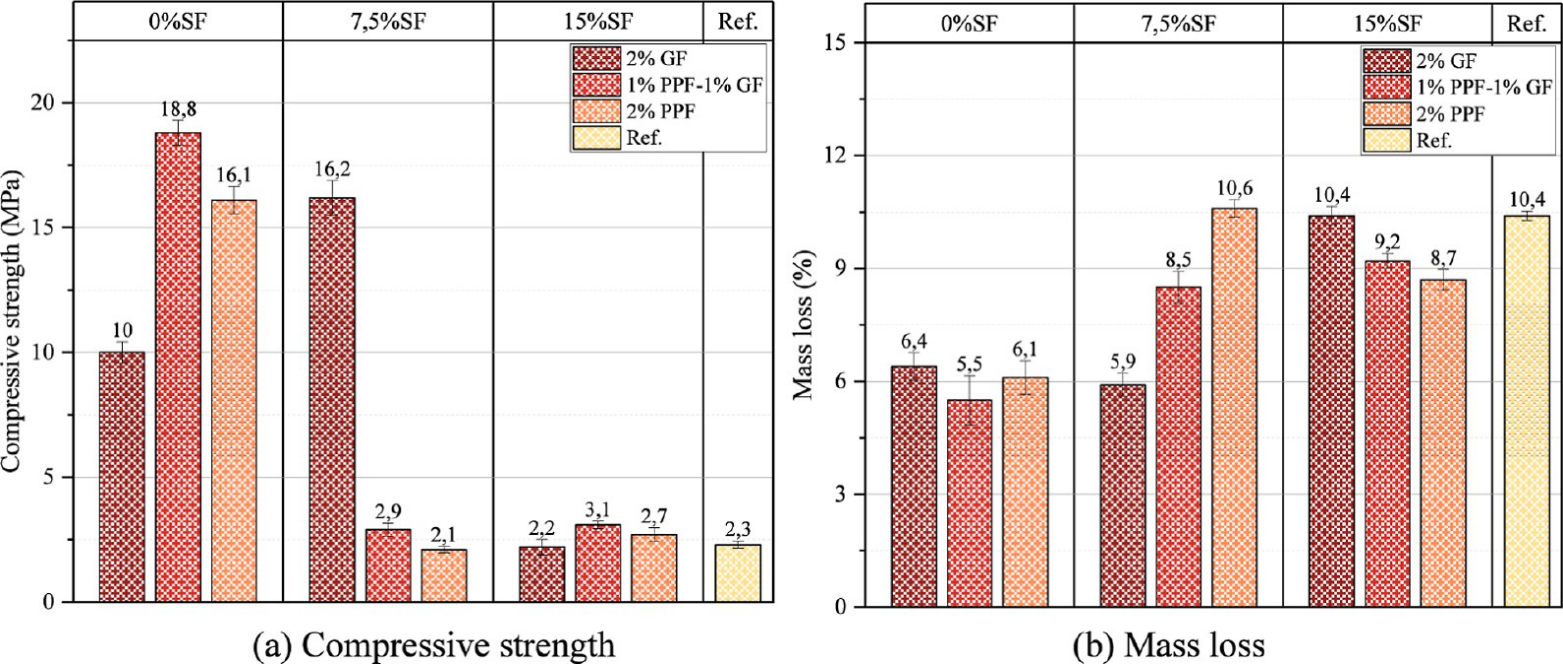
Compressive strength and mass loss after freeze–thaw test.

The increase in the amount of SF in the mixture also caused an increase in the mass loss after the freeze–thaw test. The reason for this situation is that the rise in the amount of SF causes the volume of water around these voids to increase and decrease depending on the increase in the number of voids in the matrix. If 15% SF is used, the mass loss increases to 8.7% (Figure 12b). Regarding freezing-thawing, using SF at an optimum rate in foam geopolymer concretes is essential. In comparing the reference sample with other samples, the compressive strength after freezing-thawing was calculated as 2.3 MPa and the mass loss as 10.4%.

#### 3.6.2 High-Temperature Resistance

The compressive strengths of the samples after the high-temperature effect of 200, 400 and 600 °C are presented in Figure 14. The use of glass and polypropylene fibers in the samples without SF primarily increased the compressive strength of the samples exposed to 200 °C (Figure 13a). At the high-temperature value of 200 °C, glass and polypropylene fibers prevented the structural integrity of the matrix from deteriorating. Glass fibers preserved their structural properties at 200 °C and prevented the formation of cracks caused by thermal stresses in the matrix. Polypropylene fibers melted at 200 °C and formed voids in the concrete matrix. These voids caused the heat to spread more homogeneously. The increase in the mixture’s SF ratio also caused the samples exposed to 200 °C compressive strength to decrease. However, 200 °C is a relatively low temperature, and microscopic voids formed in the matrix due to SF in the foam geopolymer concrete at this temperature. These voids increased in microscopic size due to thermal stresses. The increased void volume also caused a decrease in structural integrity. An increase in compressive strength was observed in samples exposed to 400 °C when compared with the 28 day compressive strength results (Figure 13b). However, these increases differed according to the SF and fiber combination in the mixture. An increase in compressive strength was observed, especially in samples without SF after 400 °C. The geopolymerization process in the geopolymer matrix continued with the effect of high temperature. In particular, the chemical reactions of the matrix’s reactive substances, such as silica and alumina, continued under high temperatures. These chemical reactions also caused an increase in compressive strength. In addition, the C–S–H gel became more stable under the effect of high temperature. The calcium-silicate bonds in the C–S–H gel became stronger with the increase in temperature. As the temperature increased, the chemical bonds between calcium and silica became more substantial, and the structure of the C–S–H gel became more solid. The crystallization processes in the internal structure of the C–S–H gel also formed a more regular and solid crystal structure at high temperatures. These crystal structures also caused the compressive strength of the samples to increase after the high-temperature effect of 400 °C. Although the C–S–H gel is generally amorphous, it can form more regular crystal structures at high-temperature ranges (such as 200 and 400 °C). Another reason is that the effectiveness of the hydrogen (H) bonds in the C–S–H structure plays an important role. At high temperatures of 200 and 400 °C, the H bonds were rearranged and, therefore, caused the compressive strength to increase.

**Figure 14:**
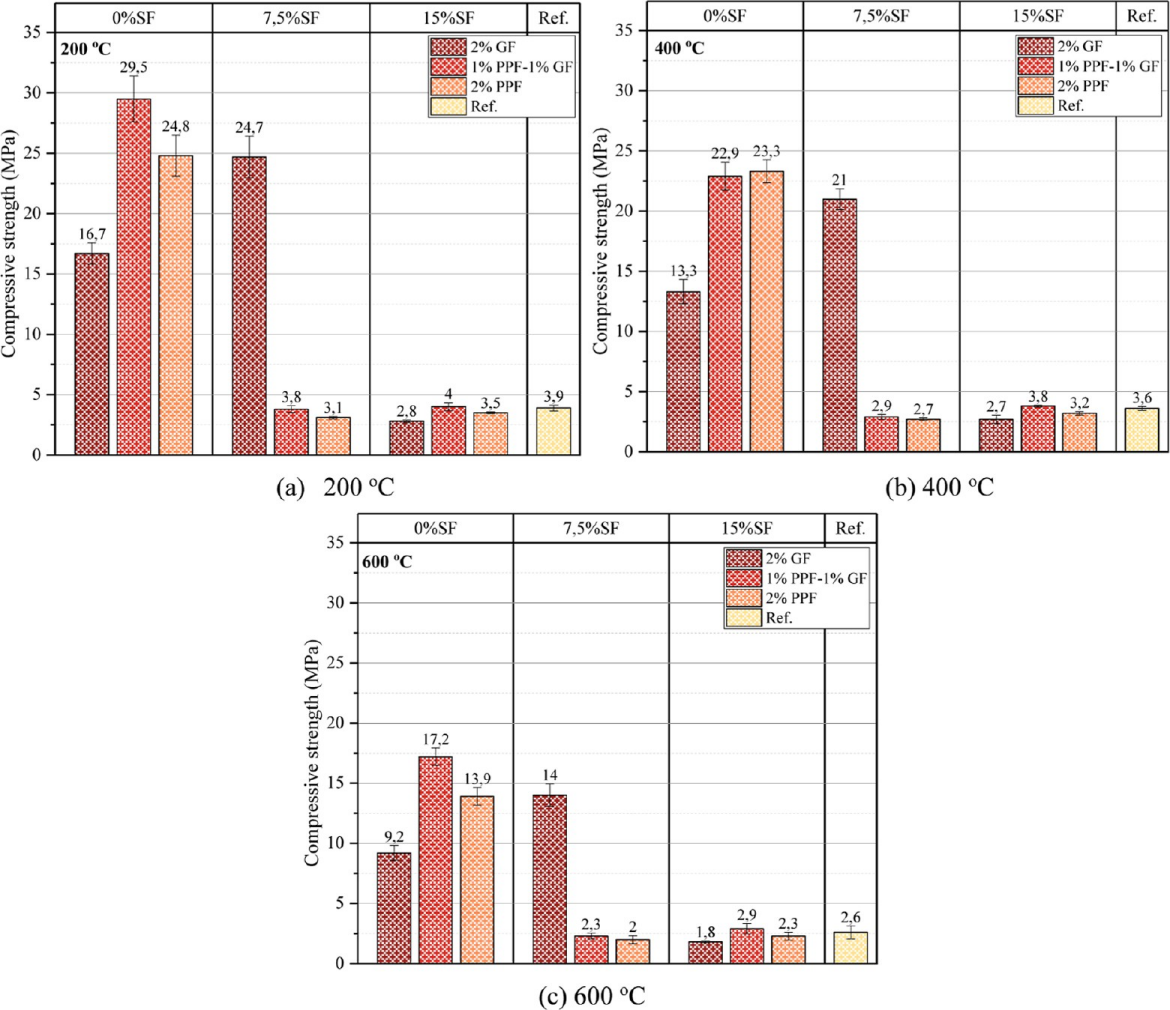
Compressive strength of tested samples after high temperature (a) 200 (b) 400 (c) 600 °C.

When the samples were exposed to a temperature of 600 °C, there was a decrease in the compressive strength (Figure 13c). The size of the microcracks in the matrix may have increased due to thermal stresses under the high-temperature effect of 600 °C. In this case, it caused the compressive strength to decrease. However, the water in the matrix may have evaporated under the high-temperature effect. In place of the evaporated water, voids were formed. These voids caused an increase in the number of pores in the matrix. The increase in the number of pores also increased the porosity. The increased porosity also caused a decrease in the compressive strength. In addition, the bonds in the C–S–H gel were damaged under a high temperature of 600 °C. The damaged bonds also caused a decrease in the compressive strength. The kinetic energy of the atoms and molecules also increased with the increasing temperature. The increase in kinetic energy increased the vibrations and caused the distance between the atoms. Since the distance between the atoms increased, the strength of the bonds in the C–S–H gel also weakened.

The mass loss of tested samples after high temperature is shown in Figure 15. The mass loss at 200 °C varies between 4% and 16%. The lowest and highest mass loss were obtained in the 0% SF–2% GF (sample containing only GF) and 15% SF–2% GF (sample containing only PPF), respectively. Since the thermal stability of PPF fibers is lower than GF fibers, the mass loss of PPF fiber samples is higher after 200 °C. While glass fibers remain more durable than polypropylene fibers at 200 °C, polypropylene fibers may start to melt at this temperature value, which causes an increase in mass loss. The mass loss after the high temperature at 400 °C varies between 6% and 18%. The melting of PPFs was higher at 400 °C.

**Figure 15:**
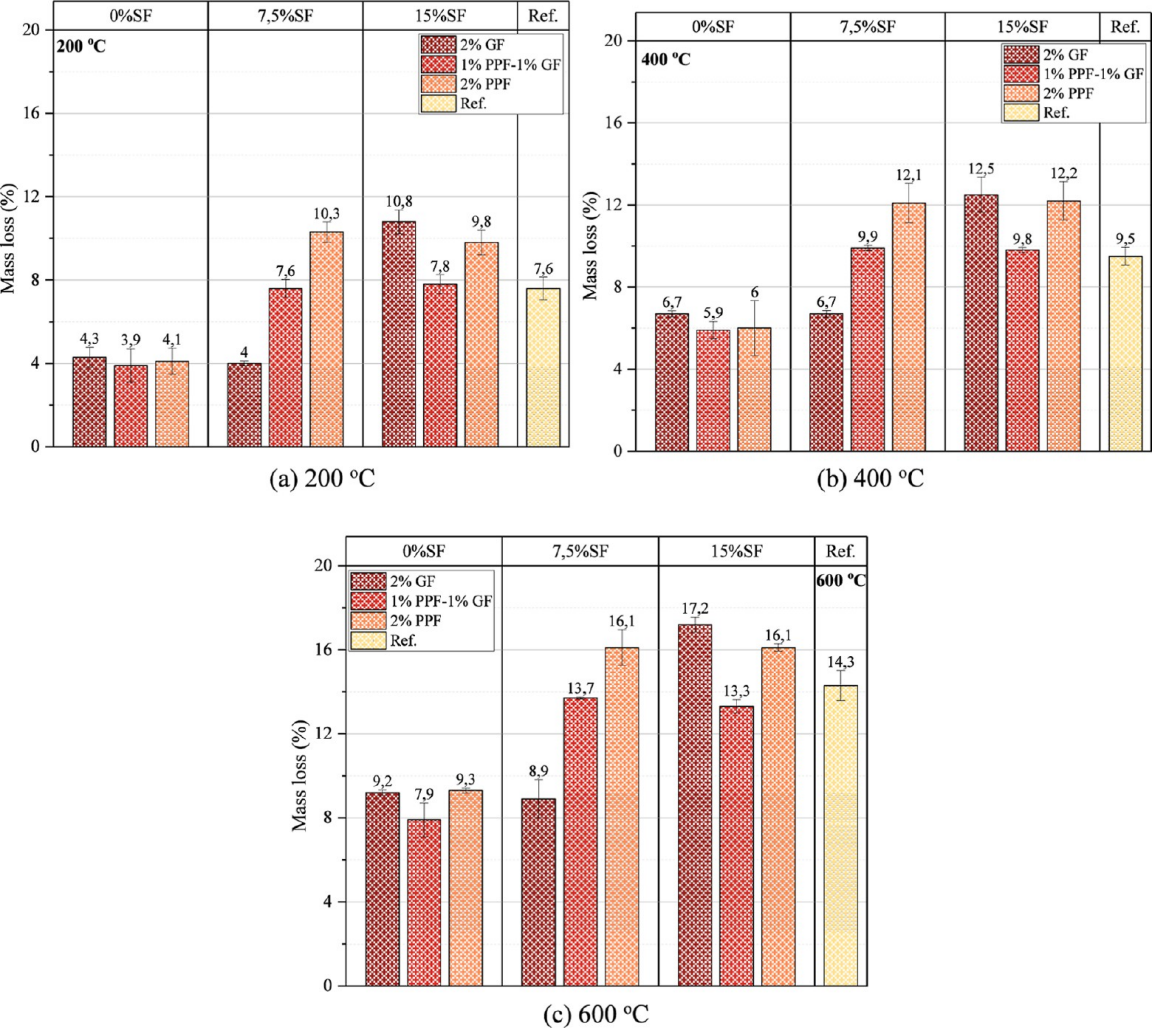
Mass loss of tested samples after high temperature (a) 200 (b) 400 (c) 600 °C.

For this reason, the mass loss increased. Again, the reason for the low mass loss of GF-added samples at this temperature value is the high-temperature resistance of glass fibers. Although SM used in the mixture increased the high-temperature resistance of the samples, an adverse effect of polypropylene fibers was observed at 400 °C. The mass loss at 600 °C varied between 9% and 20%. The fact that the PPFs were wholly melted at this temperature caused an increase in the mass loss. Sim and Park^82^ reported the effectiveness of the high-temperature resistance of glass fibers. Furthermore, they indicated that it was seen that after subjecting to 1200 C for 2 h, GF underwent partial melting. In the presented study, it was determined that GF has better high-temperature resistance compared to PPF, and this advantage is also reflected in high-temperature resistance.

The relationship between compressive strength and mass loss after the high-temperature effect is presented in Figure 16. The mass loss of the samples after high temperature provides essential information about the chemical and physical changes that occur in the internal structure of the matrix. For this reason, the relationship between compressive strength and mass loss of the samples after the high-temperature effect was emphasized. In the samples exposed to temperatures from 200 to 400 °C and then to 600 °C, a decrease in compressive strength and an increase in mass loss are observed with the temperature rise. The reduction in the density of the samples with the increasing temperature increased the void ratio in the internal structure and caused the compressive strength to decrease. Especially in the fibrous or hybrid fiber samples (especially in PPF samples) exposed to a temperature of 600 °C, the melting of the fibers at this temperature value also caused the mass loss to increase. The increased mass loss also caused the compressive strength to decrease.

**Figure 16:**
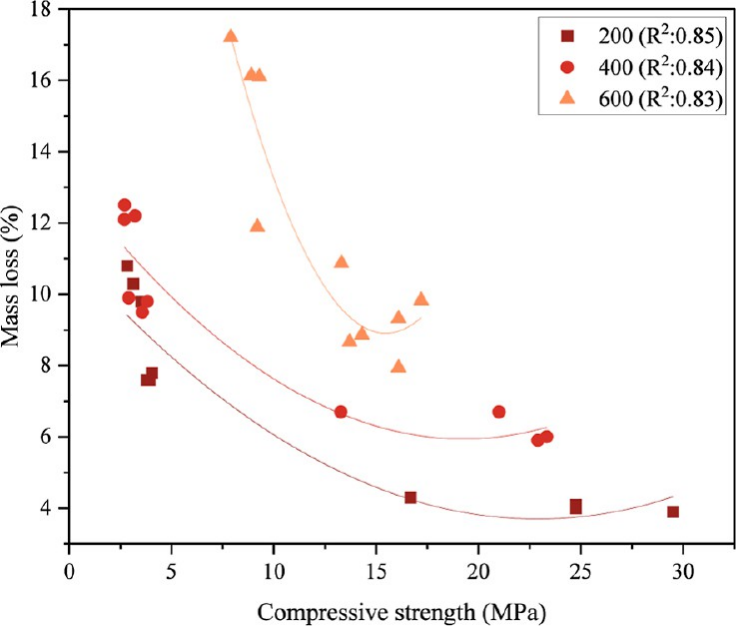
Relationship between mass loss and compressive strength after high temperature.

The *R* ^2^ values in the graph also show the importance of the relationship between mass loss and compressive strength. The fact that the *R* ^2^ value is more significant than 0.8 for all three temperature values indicates a strong relationship between mass loss and compressive strength and a strong correlation between these two variables. Although there is a decrease in the relationship between compressive strength and mass loss with increasing temperature (*R* ^2^ = 0.85 for 200 °C, *R* ^2^ = 0.84 for 400 °C and *R* ^2^ = 0.83 for 600 °C), this decrease is low. The fact that *R* ^2^ = 0.85 for 200 °C shows that the change in mass loss at this temperature value significantly affects the reduction in the compressive strength of the samples. Since GF remains stable at 200 °C and maintains its compressive strength, this relationship may be more pronounced in mixtures containing GF. *R* ^2^ = 0.84 for 400 °C shows the strong relationship between mass loss and compressive strength continues. However, a slight decrease is observed in the *R* ^2^ value. This situation may be due to the reduction in mass loss due to the melting of PPF fibers at 400 °C.

Nevertheless, since the strong relationship between mass loss and compressive strength continues at 400 °C, the effect of deterioration in the material structure on compressive strength is essential. At 600 °C, despite the *R* ^2^ value decreasing to 0.83, the mass loss-compressive strength relationship is still strong. The reason for this situation is that the PPF fibers completely melt at 600 °C, and the mass loss increases due to the decrease in thermal stability of other materials at this temperature. However, the relationship weakens slightly as the material’s thermal stability weakens. Another reason for this situation is that material deterioration is more complex at 600 °C.

### 3.7 Microstructure Studies

In the image, an inhomogeneous microstructure is observed (Figure 17a). This shows some voids and weak regions within the binder phase (geopolymer matrix). In the reference mix, it is clear that the binding quality is limited, primarily due to the absence of silica fume (SF) or fiber additives, which leads to a loose microstructure. Weak areas are in the form of larger voids or microcracks seen under a microscope. These voids result from unreacted particles in the matrix or insufficient polymerization. The apparent porosity of the reference blend (Figure 5a) is as high as 29.1%. This ratio is directly related to the loose structure in the SEM image. The high void ratio indicates the matrix has a lower density (1385 kg/m^3^, Figure 5c). The 28 day compressive strength of the reference mix (Figure 6b) is only 2.6 MPa. This low value indicates that the matrix does not have a dense structure and the polymerization reaction is incomplete. The microcracks and inhomogeneous bond structure observed in the SEM image explain this low strength. The reference mixture’s loose microstructure and high porosity are the main reasons for the poor mechanical properties. The limited reaction of the alkaline activator (sodium metasilicate) and binder (GBFS) phase results in less density. The use of SF additives was insufficient to fill the microvoids in the matrix and maintained a weak bond system, so this increased mechanical strength did not change. SEM images correlated well with the macroscopic results and characterized the reference mix’s poor mechanical properties and large porosity. This poor microstructure is caused by fewer reacted particles and inhomogeneous polymerization.

**Figure 17:**
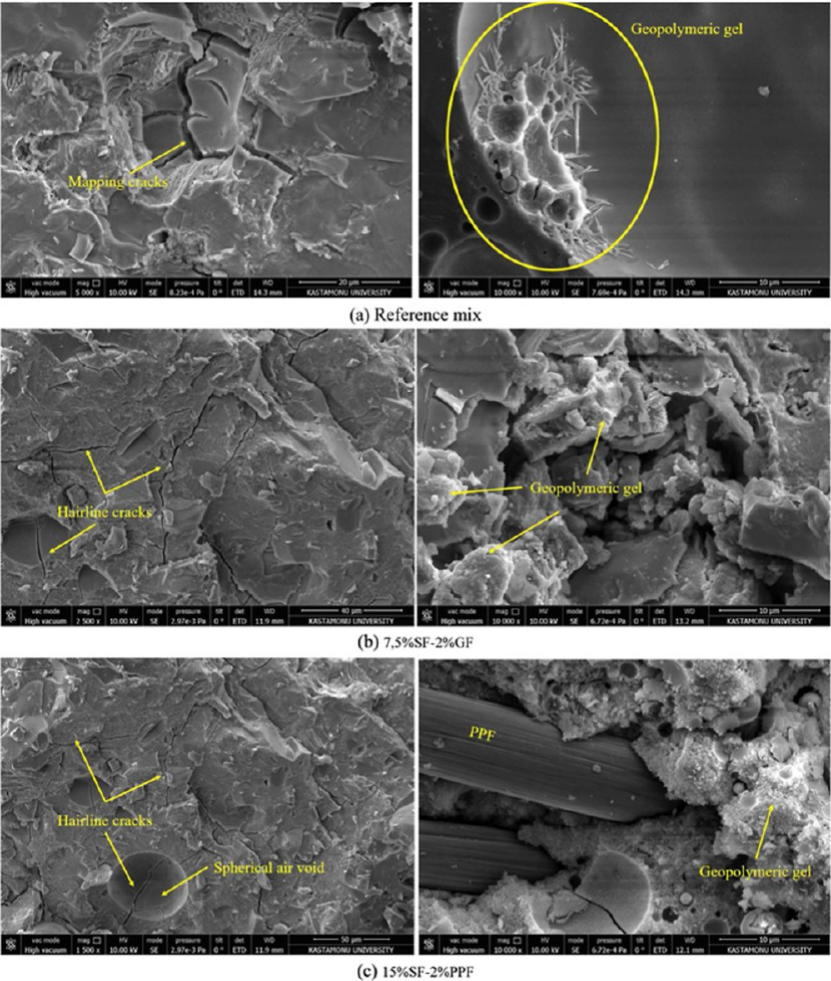
SEM images of geopolymer foams.

The SEM image reveals a much denser microstructure compared to the reference mix (Figure 17b). Silica fume (SF) and glass fiber (GF) additives increased the filling ratio of the binder phase and reduced the microvoids. Although irregularly distributed microcracks and voids are still observed in the matrix, they are reduced in size and density. This is related to SF’s pozzolanic effect and GF’s homogeneous bond structure within the matrix. It is understood that silica fume acts as an effective filler and reacts in the matrix due to its fine-grained structure and high reactivity. This increases both the mechanical properties and density of the matrix. The apparent porosity of the mixture containing 7.5% SF and 2% GF is considerably lower than the reference mixture (14.2%, Figure 5a). The observation of fewer voids in the SEM image supports this conclusion. The lower porosity increased density (about 1685 kg/m^3^, Figure 5c). The 28 day compressive strength of the 7.5%SF-2%GF mixture was 19.1 MPa (Figure 6b). This value is about 7.3 times higher than the reference mix (2.6 MPa).

The SEM image reveals an irregular microstructure with highly pronounced and large voids within the matrix (Figure 17c). This indicates that the 15% SF content is not homogeneously distributed within the matrix and that the excessive use of SF leaves unreacted particles in the binder phase. The void areas also originated from weak bonding within the uniaxially oriented cutting pattern provided by polypropylene fibers (PPF) because PPF has low tensile strength and less rigid structure inside these voids. The distribution of PPF in the matrix was observed using an SEM image. These fibers restrict the propagation of microcracks and have a small amount in bond structure. Unfortunately, due to PPF having a low elastic modulus and high elongation at the break, the formation of microvoids in the matrix could not be avoided entirely.

Furthermore, the density of the blend was found to be low (1382 kg/m^3^, Figure 4c), indicating the loose nature of the matrix and the negative effect of overutilization of SF. The 28 day compressive strength of this mixture was only 3.4 MPa (Figure 5b). This low value was due to the weak bonding structure of the microstructure seen in the SEM image and the unreacted particles formed due to excessive use of SF. A high proportion of SF, as high as 15%, formed extremely dense and unreactive regions in the matrix. The visible large pores in the SEM image indicate this. An extremely high SF restrains the polymerization, disrupts the bond structure, and weakens the mechanical properties. Though PPF itself has the potential to restrict microcrack advancement, its low tensile strength and stiffness were insufficient to raise matrix density. SEM image showed that the PPF dispersed loosely in the matrix, resulting from a less effective bond structure.

## 4 Conclusions

This study aims to experimentally investigate the effects of silica fume ratio (0%, 7.5% and 15%) and glass and polypropylene fibers on foam geopolymer concrete’s mechanical, physical, durability, and microstructure. Within the scope of the experimental study, physical properties of flow diameter, sorptivity, water absorption and oven-dry density were determined. In addition, compressive strength, flexural strength, and thermal conductivity coefficient parameters were investigated. The effects of silica fume and hybridization of glass and polypropylene fibers on freeze–thaw and high-temperature performance and microstructure were also investigated experimentally. The following findings were achieved in summary due to the experimental study. The experimental results obtained within the scope of the study are valuable, especially in producing environmentally friendly foam geopolymer concrete using sustainable materials. However, it was concluded that pozzolanic materials such as silica fume should be used carefully; otherwise, they may negatively affect the mechanical strength. At the same time, although using glass fiber and polypropylene fiber together positively affected the strength, this effect became more pronounced in mixtures that did not contain silica fume.

- The mixture and experimental results in the table are valuable mainly for producing environmentally friendly geopolymer concretes using sustainable materials. However, it is understood that pozzolanic materials such as SF should be used carefully; otherwise, they may negatively affect the mechanical strength. At the same time, the combined use of GF and PPF has a positive effect on the strength, and this effect became more pronounced in mixtures without SF. SF has a similar effect on flexural strength to compressive strength. Excessive use of SF also hurts flexural strength.
- The use of sodium metasilicate in foam geopolymer concrete caused the activation of the matrix to gain early strength. However, using high levels of silica fume increased the matrix’s density and reduced sodium metasilicate’s effect on early strength. Sodium metasilicate gave more practical results at low levels of silica fume and increased flexural and compressive strength by improving the fiber–matrix interaction.
- When the effect of fiber additives on peak load and peak displacement was evaluated, it was observed that the mixtures containing 2% glass fiber reached the highest peak load value in mixtures without SF. In comparison, combining 1% glass fiber and 1% polypropylene fiber increased load-carrying capacity and deformation capability.
- Using silica fume and GF and PPF fiber combination in optimum proportions decreased the thermal conductivity of foam geopolymer concrete and increased the insulation performance. Both silica fume and fiber addition increased the density of the matrix, resulting in a decrease in thermal conductivity.
- In mixtures not containing silica fume, especially in samples reinforced with hybrid fibers (GF and PPF), the loss of compressive strength after the high-temperature effect is low. Increasing the silica fume ratio caused a significant decrease in compressive strength at high temperatures. Severe strength loss was observed under high temperatures, especially in samples containing 15% silica fume. Although the role of sodium metasilicate as an activator is essential, it can negatively affect the microstructure when used together with SF. Strength can be increased by the balanced use of silica fume and sodium metasilicate and the reinforcement of hybrid fibers in foam geopolymer samples exposed to high temperatures.
- The use of glass fiber in the mixtures caused the mass loss of the samples exposed to high temperatures to decrease. Polypropylene fibers, on the other hand, caused the mass loss to increase, especially since they started to melt at temperatures of 400 °C and above.
- It was determined that there is a strong correlation between mass loss and compressive strength. The *R* ^2^ value decreased with increasing temperature. However, this decrease was not at significant levels. This decrease was caused by the melting of polypropylene fibers at high temperatures.
- SEM analyses showed that SF and fiber additives significantly affected the microstructure of the geopolymer foams compared to the reference blend. The blend with 7.5% SF and GF provided a more dense and homogeneous microstructure, reducing the void ratio and improving the mechanical strength. However, due to excessive SF usage, the blend containing 15% SF and PPF showed high porosity and unreacted zones, leading to poor mechanical properties.
- This study demonstrates the potential of geopolymer foams for energy-efficient structures due to their lightweight and thermal insulation properties. However, the SF ratio and fiber type should be carefully selected in applications with critical mechanical strength.
- In this study, the effects of different silica fume ratios and fiber combinations on the performance of foam geopolymer concrete were investigated in detail. Considering the mechanical, thermal and durability properties, 0% SF–1% GF/1% PPF blend gave the best results. This study has shown that SF can have both positive and negative effects on the performance of the mix. Using SF in low proportions (e.g., 7.5%) improved the microstructure by increasing the density of the mixture and keeping its viscosity under control. However, using high proportions of SF (15%) generally caused unfavorable results.
- An environmentally friendly concrete alternative was developed using waste materials (e.g., waste marble powder and slag). In the future, it would be helpful to carry out studies on the usability of such materials in large-scale industrial applications. This study was carried out under specific laboratory conditions. In the future, it is recommended that the behavior of the specimens be evaluated under different ambient conditions (e.g., humidity, corrosive environments).
- In this study, only certain silica fumes (0%, 7.5%, 15%) and fiber ratios (GF and PPF) were tested. Different results can be achieved with a broader range of materials and mixtures. Freeze–thaw and high-temperature tests were conducted to evaluate short-term performance. However, the long-term consequences of these effects (e.g., between 1 and 5 years) have not yet been investigated. This study was carried out on a laboratory scale. The behavior of such concretes in large-scale field applications (e.g., large building projects) has not yet been tested.
